# Supplementary material for: Seeing the invisible: How RNA extraction and library prep shape viral read recovery in RNA-seq
Source: Mol Ther Adv. 2026 Oct 6;34(4):201858. doi: 10.1016/j.omta.2026.201858 (PMC13718637; doi:10.1016/j.omta.2026.201858)
Supplement: Supplementary file 1 [file mmc1.pdf]

OMTA, Volume *34*

Supplemental information

Seeing the invisible: How RNA extraction and library prep shape viral read recovery in RNA-seq

Sayma Afroj, Binsheng Gong, Aaron Scholl, Bingjie Li, Joshua Xu, and Sandip De

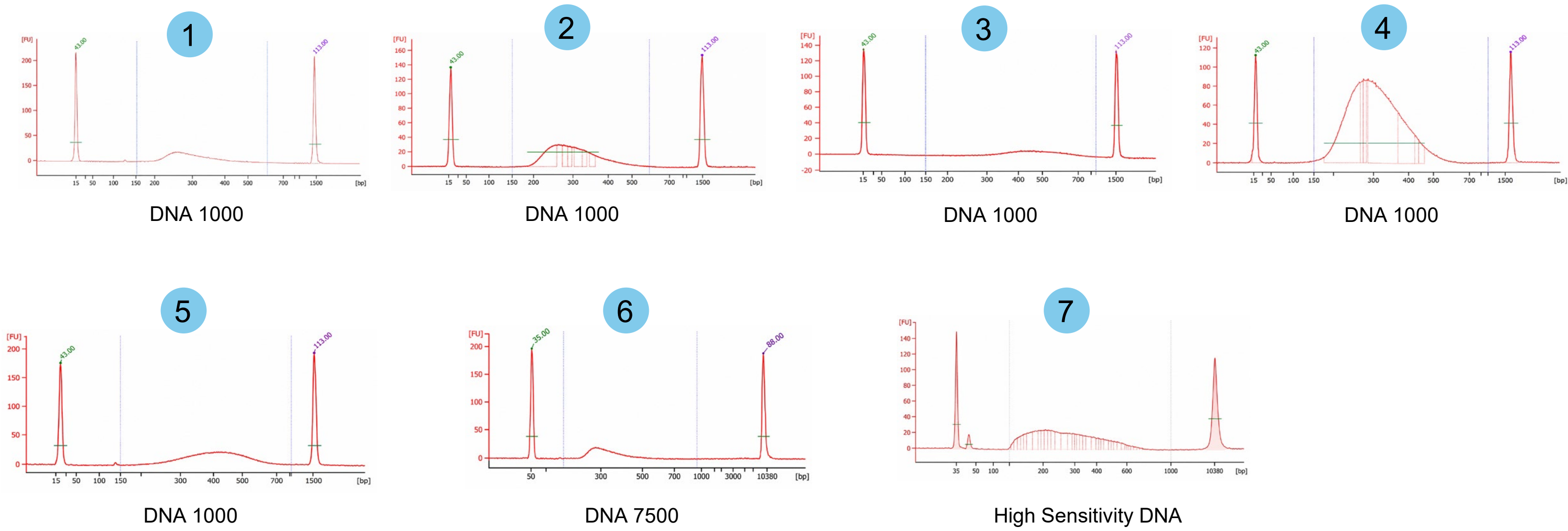

Fig S1. Representative electropherogram images for RNA-Seq libraries prepared using 7 different methods. Bioanalyzer DNA kits used for analyzing the library distribution is mentioned below.

Group		HEV-AVG	HEV-SE	RSV-AVG	RSV-SE	REO-AVG	REO-SE	ZIKV-AVG	ZIKV-SE	FeLV-AVG	FeLV-SE
1	Control	0.000	0.000	0.062	0.022	0.013	0.013	*15.403*	*3.671*	0.000	0.000
	Cocktail 1	0.191	0.037	2.946	0.027	0.271	0.071	*15.837*	*2.546*	0.060	0.005
	Cocktail 2	1.538	0.072	38.310	0.380	5.066	1.406	*37.308*	*2.555*	7.405	0.504
2	Control	0.000	0.000	0.022	0.013	0.000	0.000	*0.710*	*0.061*	0.013	0.004
	Cocktail 1	0.005	0.005	0.178	0.006	0.017	0.017	*0.781*	*0.132*	0.005	0.005
	Cocktail 2	0.132	0.009	3.075	0.010	0.251	0.022	*1.910*	*0.100*	0.993	0.035
3	Control	0.000	0.000	0.000	0.000	0.000	0.000	0.000	0.000	0.000	0.000
	Cocktail 1	0.186	0.070	7.170	0.110	0.138	0.003	0.525	0.199	0.152	0.075
	Cocktail 2	3.266	0.052	117.872	6.174	5.358	0.035	21.485	1.473	26.225	0.686
4	Control	0.062	0.062	1.569	1.465	0.118	0.100	1.014	0.815	0.473	0.473
	Cocktail 1	*0.000*	0.000	37.879	4.426	0.136	0.136	0.885	0.367	0.141	0.141
	Cocktail 2	*0.000*	0.000	658.374	11.662	1.078	0.057	5.479	2.407	2.419	0.086
5	Control	0.004	0.004	0.128	0.128	0.000	0.000	0.000	0.000	0.000	0.000
	Cocktail 1	0.013	0.000	54.850	17.272	0.007	0.007	0.562	0.550	*0.000*	0.000
	Cocktail 2	0.011	0.011	624.347	27.601	0.633	0.375	1.141	0.364	5.015	2.135
6	Control	0.000	0.000	0.000	0.000	0.012	0.001	*31.068*	*2.549*	0.000	0.000
	Cocktail 1	0.387	0.125	4.490	0.063	0.419	0.031	*27.348*	*4.926*	0.141	0.053
	Cocktail 2	2.723	0.219	49.944	3.463	4.099	0.394	*67.402*	*30.723*	16.322	1.700
7	Control	0.000	0.000	0.000	0.000	0.000	0.000	0.000	0.000	0.000	0.000
	Cocktail 1	0.048	0.048	1.178	0.431	0.024	0.024	0.127	0.042	0.023	0.001
	Cocktail 2	0.568	0.202	26.279	2.010	1.209	0.043	2.696	0.343	4.721	0.982

Fig S2. Mapped read counts (CPM) for HEV, Reovirus, RSV, ZIKV and FeLV across library prep conditions and spike-in groups (Control, Cocktail 1, Cocktail 2). Each library preparation method was performed in duplicate (n = 2). Cases where the standard error equals the mean (highlighted with blue boxes) arise when one replicate has a zero value and the other contains the full signal highlighting signals in control samples are stochastic single-replicate events.

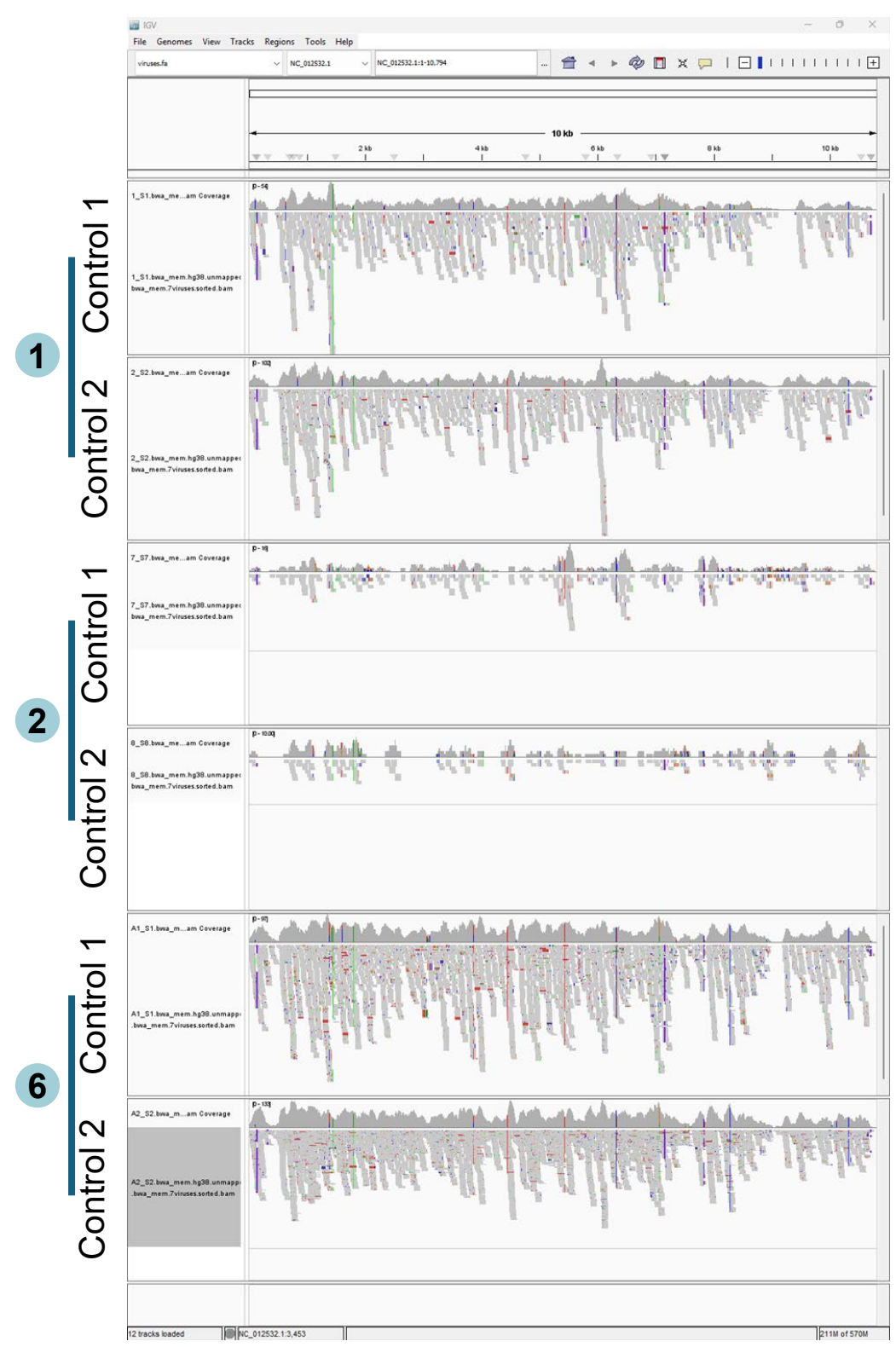

Fig S3. An Integrative Genomics Viewer (IGV) screenshot showing BAM file alignments from control samples generated using library preparation methods 1, 2, and 6.

Tables S1–S6

Table S1. RT-qPCR CT values for comparison of relative viral RNA recovery across RNA extraction methods and sample matrices. CT values for the indicated viruses following RNA extraction using the five evaluated methods. Ct values were used as comparative measures of relative viral RNA recovery within the same virus and sample matrix and were not used for absolute quantification or determination of extraction efficiency.

Table S2. Initial exploratory mapping analysis- RNA-seq read mapping results across library preparation methods and experimental conditions. We used a combined host + viruses genome for the analysis.

Table S3. Final host-filtered viral mapping- RNA-seq read mapping results across library preparation methods and experimental conditions. We used a combined host - viruses genome for the analysis.

Table S4. Competitive flavivirus re-alignment analysis of ZIKV-mapped reads detected in unspiked control samples. ZIKV-mapped reads from control samples were re-aligned against an expanded panel of closely related flaviviruses using paired-end and independent single-end alignment strategies to assess whether the unexpected ZIKV signal resulted from misclassification.

Table S5. Untargeted viral detection using the Reference Viral Database (RVDB), post host read filtering. Summary of viruses identified by the RVDB-based bioinformatics pipeline across library preparation methods and experimental conditions. Results represent screening-level bioinformatic hits and should not be interpreted as confirmed viral detections without additional confirmatory evaluation.

Table S6. RNA-seq library preparation and sequencing batch information. Summary of library preparation and sequencing batch information for samples sequenced using the Illumina Novaseq 6000 platform.
