# Supplementary material for: Seeing the invisible: How RNA extraction and library prep shape viral read recovery in RNA-seq
Source: Mol Ther Adv. 2026 Oct 6;34(4):201858. doi: 10.1016/j.omta.2026.201858 (PMC13718637; doi:10.1016/j.omta.2026.201858)
Supplement: Supplementary file 2 [file mmc8.pdf]

Molecular Therapy
Advances
Original Article

Seeing the invisible: How RNA extraction and library prep shape viral read recovery in RNA-seq

Sayma Afroj,[1] Binsheng Gong,[2] Aaron Scholl,[1] Bingjie Li,[1] Joshua Xu,[2] and Sandip De[1]

[1]Tumor Vaccine and Biotechnology Branch, Division of Cellular Therapy 2, Office of Cellular Therapy and Human Tissue, Center for Biologics Evaluation and Research, U.S. Food and Drug Administration, Silver Spring, MD 20993, USA; [2]Division of Bioinformatics and Biostatistics, Office of Research, National Center for Toxicological Research, U.S. Food and Drug Administration, Jefferson, AR 72079, USA

Next-generation sequencing (NGS) is emerging as a powerful tool for detecting adventitious viral agents in biologics, aligning with recommendations from ICH Q5A(R2). However, the performance of RNA-seq-based detection assays is highly dependent on upstream sample preparation steps and data analysis. In this study, we systematically evaluated five RNA extraction methods and seven RNA-seq library preparation protocols across multiple sample matrices spiked with model viruses. Our results demonstrate variability in viral RNA recovery depending on extraction chemistry, virus, and sample matrix type. Tri reagent-based methods excelled for certain viruses, while magnetic bead-based extraction yielded the highest total RNA in most matrices tested. Among RNA-seq library prep methods, rRNA depletion post-library construction combined with dual indexing improved viral signal detection and minimized artifacts such as index hopping. Finally, we implemented a custom bioinformatics pipeline to enhance sensitivity and minimize host-background interference. These findings highlight critical analytical variables that influence NGS assay performance and provide practical guidance for optimizing workflow components to support the use of NGS for adventitious agent detection in cell and gene therapy product development.

INTRODUCTION

The detection and characterization of pathogens and adventitious agents in biological products derived from human or animal cells is a critical component of product safety evaluation. Traditional adventitious viral agents testing (AAT) methods (*in vitro*/*in vivo* assays, electron microscopy, etc.) have several drawbacks: (1) standard *in vitro* and *in vivo* assays can take several weeks (e.g., 28 days) to complete, which is not ideal for expedited product development timelines, especially in personalized or rapidly manufactured therapies, (2) *in vitro* assays depend on observable cytopathic effects (CPEs) or viral replication, sometimes making them insensitive to non-replicating, or non-cytopathic viruses, (3) *in vivo* testing relies on animal models (e.g., suckling mice and embryonated eggs), raising ethical concerns and increasing operational burdens, (4) they often fail to identify novel, emerging, or unexpected agents, (5) traditional AATs do not generate molecular or genomic data, preventing detailed characterization or confirmation of agent identity, which can hinder root-cause investigations, (6) multiple orthogonal assays must be used to cover a broad spectrum of adventitious agents, resulting in increased cost, complexity, and sample volume requirements, and (7) assays originally developed for simpler biologics (e.g., monoclonal antibodies and vaccines) may not be directly suitable for complex matrices such as cell and gene therapy products.

Next-generation sequencing (NGS) is an agnostic approach which allows rapid, sensitive, and specific detection of pathogens and adventitious agents without any prior knowledge.[1–3] Following the release of the ICH Q5A(R2) guidance and supporting reports,[4–11] the adoption of NGS for viral and microbial safety assessment has accelerated. NGS is a complex process, optimizing each step of the NGS pipeline-from nucleic acids extraction to library preparation and bioinformatic analysis is essential to ensure sensitivity and specificity.[5,6,12–14] Subsequently, European Directorate for the Quality of Medicines & HealthCare (EDQM) published the new general chapter 2.6.41 on “high-throughput sequencing for the detection of viral extraneous agents” in Edition 12.2 of the European Pharmacopoeia (Ph. Eur.) in October 2025, this new chapter defines expectations for a well-validated NGS assay.[15] Despite its high-throughput and agnostic potential, the performance of NGS can be significantly influenced by technical variables.

Challenges such as inefficient extraction of viral RNA from complex matrices, low abundance of viral sequences in host-rich samples, and artifactual signal amplification require methodical optimization. Notably, recent documents have emphasized the importance of

Received 18 February 2026; accepted 1 October 2026;
https://doi.org/10.1016/j.omta.2026.201858.

Correspondence: Sandip De, Tumor Vaccine and Biotechnology Branch, Division of Cellular Therapy 2, Office of Cellular Therapy and Human Tissue, Center for Biologics Evaluation and Research, U.S. Food and Drug Administration, Silver Spring, MD 20993, USA.
E-mail: sandip.de@fda.hhs.gov

This is an open access article under the CC BY license (http://creativecommons.org/licenses/by/4.0/).

matrix-specific evaluation of viral RNA extraction protocols, where "matrix" refers to the biological sample type or background being tested[4,16] and the variable impact of different RNA-seq library preparation kits on viral read recovery,[16–18] particularly when applied to clinical or production-relevant cell lines.

Given the known effects of virus type, sample matrix, and workflow design on NGS assay performance, we systematically evaluated a combination of viruses and biologically relevant matrices to identify variables influencing viral RNA recovery and detection. We assessed five RNA extraction methods across three sample matrices-HEK293 cells (commonly used for adeno-associated virus production), U937 cells, and human buffy coat samples-using qRT-PCR to measure viral RNA recovery. To provide a biologically relevant reference material for assay qualification, we also established and characterized a persistently ZIKV-infected U937 monocyte cell line as a model relevant to cell-based therapeutic products. Characterization of this cell line revealed sequence-level mutations that accumulated during long-term viral replication compared with the reference genome.

We further compared seven RNA-seq library preparation strategies for their ability to detect spiked viral RNAs in human cell backgrounds. To improve the accuracy of viral detection and minimize false positives, we applied a customized bioinformatics pipeline designed to prefilter host-derived reads before viral mapping. This approach was essential, as initial analyses without host read removal revealed a hepatitis E virus (HEV) signal in unspiked control samples. Further investigation suggested that this signal originated from a 171-nucleotide insertion of the human ribosomal protein gene RPS17 within the HEV genome, a finding consistent with prior literature and one that highlights the importance of rigorous host-read filtering in NGS-based pathogen detection workflows.[19,20] Together, these results underscore the matrix and virus-specific performance variability of RNA extraction and library preparation methods and emphasize the need for thorough qualification and validation of each step in NGS-based viral detection workflows.

RESULTS

Generation and characterization of cells persistently infected with ZIKV as a reference material

U937 cells were infected with the ZIKV MR766 strain and cells were passaged until all cells stained positive for Zika virus (ZIKV) E protein by immunofluorescence using a pan-flavivirus antibody (Figures 1A and 1B).[21,22]

Given the dynamic and genetically heterogeneous nature of RNA viruses, which enables rapid adaptation to selective pressures, we sought to identify mutations accumulated in the ZIKV genome during prolonged cell culture. Total RNA was extracted from the persistently infected U937 cells and subjected to Illumina RNA sequencing. Viral reads were isolated from sequencing data for comparative analysis against the reference ZIKV genome (GenBank accession: NC_012532.1). Across two technical replicates, thirteen high-confidence mutations (allele frequency ≥95%) were consistently identified compared with the reference genome (Figure 1C).

Qualification of matrix-specific RNA extraction method is important for improved viral RNA detection

As Q5A(R2) "Viral Safety Evaluation of Biotechnology Products Derived From Cell Lines of Human or Animal Origin" highlights the importance of matrix-specific verification of an NGS method for detection of viral agents, we set out to test the impact of RNA extraction methods and sample matrices on relative viral RNA recovery. We used five different RNA extraction methods (Figure 2A) and three different sample matrices (HEK293 cells, U937 cells, and buffy coat) (Figure 2B) (see the "material and methods" section for details). The selected kits represent diverse extraction chemistries (organic, silica column, and magnetic bead) commonly used in biologics testing workflows, enabling a representative comparison across methodologies. RNA extractions were performed following the manufacturer's instructions after spiking in viruses: (1) ATCC MSA 2008 (contains reovirus 3, influenza B virus B/Florida/4/2006, human respiratory syncytial virus [RSV], ZIKV, human adenovirus 40, and human herpesvirus 5) or (2) BEI NR-59622 (contains mammalian orthoreovirus type 1 strain Lang, feline leukemia virus strain Thielen [FeLV], and human respiratory syncytial virus, strain A2) + HEV RR[23] + persistently ZIKV infected U937 cells. For ATCC 2008, although DNA viruses are present in the mixture, they were not evaluated, as they are likely degraded during DNase treatment or by other extraction kit components.

For the HEK293 matrix spiked with MSA 2008, the MB RNA extraction method yielded the highest measured total RNA quantity, while the PureLink (PL) and QIAGEN (Q) methods showed similar Qubit measurements. However, because carrier RNA was included in the QIAGEN extraction protocol, the Qubit measurement for this method includes both sample-derived and carrier RNA and therefore is not directly comparable with the total RNA yields obtained using methods without carrier RNA. Tri Reagent-PureLink (TPL) ranked fourth in performance, and the lowest RNA yield was observed with the standard Tri Reagent (T) method (Figure 2B). These trends were consistent with results obtained from HEK293 samples spiked with the BEI NR-59622+HEV RR + U937-ZIKV reference panel (Figure 2B; except for the Tri Reagent method), further supporting the observed differences in total RNA recovery.

In the U937 matrix, all extraction methods, except Tri Reagent (T), which yielded the lowest RNA concentration, performed comparably in terms of total RNA recovery (Figure 2B). For the Buffy Coat matrix, the highest RNA yields were obtained using Tri Reagent (T) and Tri Reagent-PureLink (TPL) methods. In contrast, the PL method resulted in the lowest RNA yield (Figure 2B), potentially due to suboptimal lysis of the Buffy Coat samples by the PL lysis buffer.

Reference viruses' detection by RT-qPCR

Relative viral RNA recovery across the five extraction methods was compared using one-step RT-qPCR. For the MSA 2008-HEK293

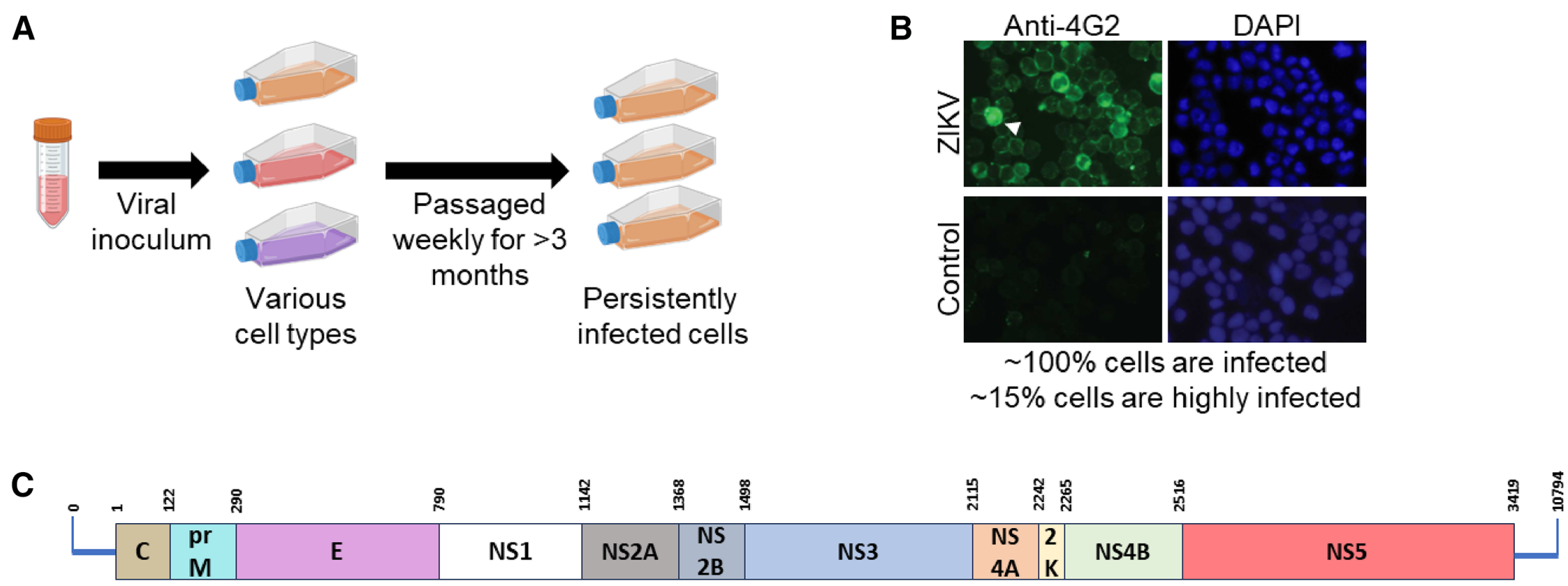

CHROM	POS	REF	ALT	U937 Persistent rep #1		U937 Persistent rep #2	
				fDP|S1	AF|S1	fDP|S2	AF|S2
NC_012532.1	5444	A	T	280	100%	566	100%
NC_012532.1	7156	A	AT	262	100%	721	100%
NC_012532.1	7165	TG	T	269	100%	758	100%
NC_012532.1	8289	G	C	873	100%	2258	100%
NC_012532.1	10331	T	C	367	100%	876	100%
NC_012532.1	1812	G	A	195	99%	498	100%
NC_012532.1	2165	C	T	142	99%	300	99%
NC_012532.1	4459	C	T	256	99%	536	98%
NC_012532.1	1913	G	C	187	99%	409	98%
NC_012532.1	137	A	AT	68	99%	131	99%
NC_012532.1	114	AC	A	78	99%	137	95%
NC_012532.1	841	C	T	122	96%	238	97%
NC_012532.1	1116	C	T	228	95%	462	99%
NC_012532.1	6507	A	G	447	74%	1089	80%
NC_012532.1	6868	G	T	254	66%	587	78%
NC_012532.1	8563	A	G	795	62%	1893	76%
NC_012532.1	3205	A	T	355	59%	764	75%
NC_012532.1	4091	G	A	123	57%	309	75%

Figure 1. Generation of ZIKV persistently infected cell lines

(A) Schematic showing workflow for establishing persistently infected U937 cell lines using ZIKV MR-766. ~1×10^6 ells were infected at MOI 1 and cultured for >3 months with weekly passaging. (B) Representative immunofluorescence images showing ZIKV E protein detection with a pan-flavivirus antibody 4G2 in persistently infected U937 cells. Further characterization of the cell line shows heterogeneity in infection levels, with ~15% of cells displaying intense staining. Cells designated as highly infected are indicated with white arrowheads. (C) RNA-seq study using persistent ZIKV U937 cell line identified 13 high-confidence mutations (allele frequency ≥95%) present in two technical replicates compared with the reference genome. The schematic diagram depicts the ZIKV reference genome.

sample, the magnetic bead-based extraction method resulted in the lowest overall CT values across the spiked viruses, consistent with greater relative viral RNA recovery under these assay conditions. Notably, although Tri Reagent yielded the lowest total RNA amount and relatively higher CT values for influenza, RSV, and ZIKV, it resulted in the lowest CT value for orthoreovirus (Figure 2C, left table).

For the MSA 2008-U937 matrix, the PureLink method resulted in the lowest CT values for Influenza virus, RSV, and ZIKV, followed by QIAGEN and the Tri Reagent-PureLink. Tri Reagent generally produced higher CT values in this matrix (Figure 2C, left table).

For the NR-59622+HEV RR+U937-ZIKV HEK293 matrix, Tri Reagent resulted in the lowest CT values for orthoreovirus 1, ZIKV, and HEV, whereas the QIAGEN method resulted in the lowest CT values for FeLV and RSV (Figure 2C, left table).

Lastly, in the MSA 2008-Buffy Coat matrix, the QIAGEN method generally showed the strongest relative viral RNA recovery based on RT-qPCR CT values, followed by Tri Reagent. RNA extraction using the magnetic bead method failed in this matrix due to bead aggregation during the extraction process (Figure 2C, left table).

Across all matrices tested, Tri Reagent consistently yielded the lowest CT values for orthoreovirus, indicating greater relative recovery/detection of orthoreovirus RNA under the tested RT-qPCR conditions. To summarize performance across viral targets and sample matrices, extraction methods were ranked according to their relative RT-qPCR performance (Figure 2C, right figure). Although Tri Reagent performed favorably for specific viruses and matrices, the QIAGEN Viral RNA Mini Kit showed the most consistent relative viral RNA recovery across the diverse conditions evaluated. These findings demonstrate that relative viral RNA recovery measured by RT-qPCR varied with extraction method, biological matrix, and virus type (Figure 2C). See Table S1 for all CT values.

Evaluation of different RNA-seq library preparation methods

To compare the performance of different RNA-seq library preparation methods for the detection of viral RNA in a human cell background, U937 cells were either used alone (control) or spiked with

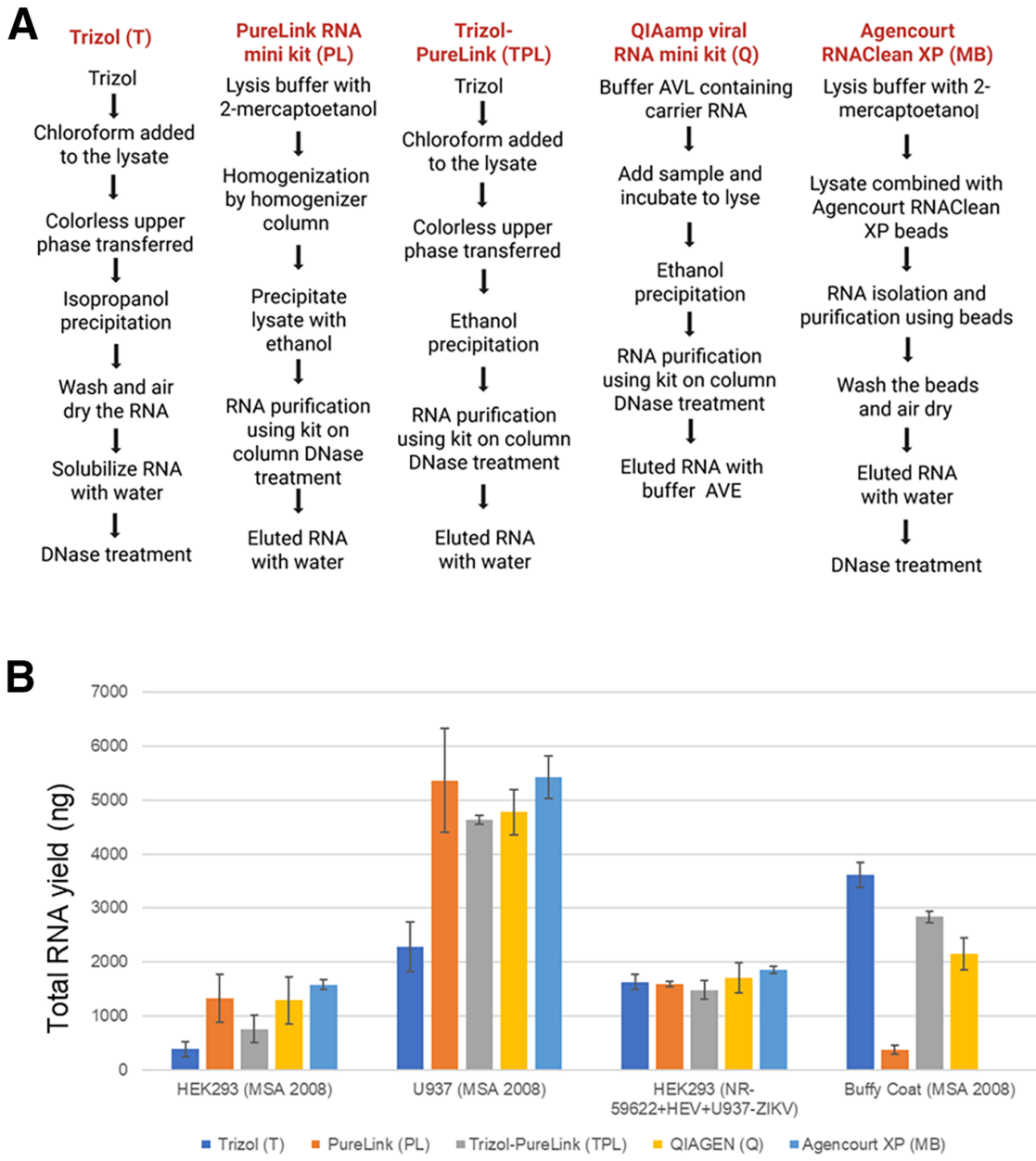

C

Viruses		RNA Extraction methods				
		T	PL	TPL	Q	MB
ATCC MSA 2008 spiked into HEK293	Reovirus 3	23.3149	29.7042	29.2931	29.7824	29.0775
	Influenza Virus	27.996	25.3252	26.0381	26.1052	24.8398
	RSV	31.3929	29.5838	30.1782	28.5242	27.1695
	Zika virus	27.7828	24.673	26.168	25.4925	24.9074
ATCC MSA 2008 spiked into U937	Reovirus 3	22.5906	29.1223	28.6572	29.6383	30.2874
	Influenza Virus	26.9054	24.9493	25.4348	25.921	26.0892
	RSV	29.8784	26.881	28.9974	27.8316	28.5095
	Zika Virus	27.8281	24.4168	25.2814	24.981	26.117
BEI NR-59622+HEV RR+U937-ZIKV spiked into HEK293	Orthoreovirus 1	23.0391	34.5628	31.8408	33.8212	35.2747
	FeLV	27.512	26.3625	26.6422	25.6449	28.2241
	RSV	26.4082	26.5498	26.3817	25.2312	27.7341
	Zika Virus	34.1603	37.2413	35.6784	34.1642	37.0031
	HEV	29.7101	32.2666	32.1542	31.09	32.6753
ATCC MSA 2008 spiked into 140 ul buffy coat	Reovirus 3	25.9029	36.0312	33.5818	33.5749	ND
	Influenza Virus	27.9903	30.2313	28.8111	28.1379	ND
	RSV	33.1956	35.833	32.3783	31.9424	ND
	Zika virus	29.2964	32.5225	29.5187	26.9886	ND

1st
2nd
3rd
4th
5th

Methods	1st	2nd	3rd	4th	5th
T	7	1	2	1	6
PL	4	2	2	8	1
TPL	0	5	9	3	0
Q	4	7	3	2	1
MB	2	2	1	3	5

CT values from qRT-PCR assays, QIAGEN showed the most consistent relative viral RNA recovery. See supplementary table 1 for more info.

Figure 2. Matrix-specific RNA extraction methods influence viral RNA recovery

(A) Diagram of tested RNA extraction methods using various cell matrices spiked with viral standards. (B) Total RNA yield across methods and matrices quantified by Qubit. Error bars represent standard error from two independent biological replicates. MSA 2008 was spiked into HEK293 or U937 cell pellets (10^5 cells) at a 1:1 virus-to-cell ratio based on viral genome copies per cell. Mammalian orthoreovirus 1, FeLV, and RSV (BEI Resources, NR-59622) were spiked into HEK293 cell pellets (10^5 cells) at the same 1:1 genome copy-to-cell ratio. ZIKV-infected cells were spiked at a 0.5:1 ratio, while HEV RR was spiked at a 0.164:1 ratio of genome copy of viruses:cells. ATCC MSA-2008 reference viral panel, containing 10^5 viral genome copies each, was spiked into 140 μL of buffy coat. (C) Left table: heatmap of qRT-PCR CT values for viral RNA across extraction methods with lowest CT value highlighted with green and highest CT value highlighted with gray, showing performance differences dependent on cell type and virus; CT values are averaged from two independent biological replicates. Right table: RNA extraction methods were ranked based on their relative performance across all tested viruses and sample matrices to identify the method with the most consistent overall performance. The ranking is intended as a summary metric and does not imply superiority for every virus or matrix. All RNA extractions were performed in duplicate (n = 2).

varying concentrations of ZIKV-infected U937 cells and a viral cocktail containing HEV and the virus panel BEI NR-59622, followed by RNA extraction and library preparation using seven different protocols, performed in duplicate (Figure 3A).

As shown in Figures 3B and S1, the fragment size distributions varied across library preparation methods. Libraries prepared with NEBNext with rRNA depletion (lane 1) and NEBNext without depletion (lane 2) showed moderately sized DNA fragments mostly ranging between 200 and 400 bp. Library prepared with Jumpcode Genomics kit showed very weak smear of DNA fragments ranging between ∼350 and 600 bp while those generated using the Trio RNA-seq kit alone (lane 4) or in combination with Jumpcode Genomics (lane 5) exhibited strong smear signals between 200 and 400 bp, indicative of successful library construction. The Ribo-Zero Plus-NEBNext Ultra II (lane 6) and RepliG-Nextera XT (lane 7) also yielded libraries, but with weaker signals and more limited size distributions.

Quantification of library yield (Figure 3C) demonstrated substantial variation among the different methods. The highest yield was obtained with the Trio RNA-seq kit alone (∼1,300 ng), followed by the Trio + Jumpcode combination (∼800 ng). Moderate yields were observed for NEBNext without or with rRNA depletion and Ribo-Zero-NEB, while Jumpcode Genomics alone, and RepliG-Nextera XT resulted in the lowest yields. Trio RNA-seq kit, likely due to its incorporation of single primer isothermal amplification (SPIA), which enhances cDNA amplification efficiency, yielded highest amount of library (Figure 3C, columns 4 and 5). In contrast, the RepliG-Nextera XT method produced the lowest yields, consistent with the Nextera XT protocol's recommendation for low DNA input (1 ng), which limits the total amount of library generated (Figure 3C, column 7). These results highlight the impact of library preparation strategy on library complexity and yield.

Filtering of host-reads is important before mapping of NGS reads to the viral genomes

We initially analyzed the processed RNA-seq reads using the exploratory bioinformatics workflow shown in Figure 4A. Following quality assessment and trimming, reads were aligned against a combined reference containing the human genome and five viral genomes (RSV, FeLV, mammalian orthoreovirus type 1, ZIKV, and HEV) using BWA-MEM (v.0.7.19).[24] Reads were assigned to the best matching genome and categorized as viral, host, or other. Unmapped reads

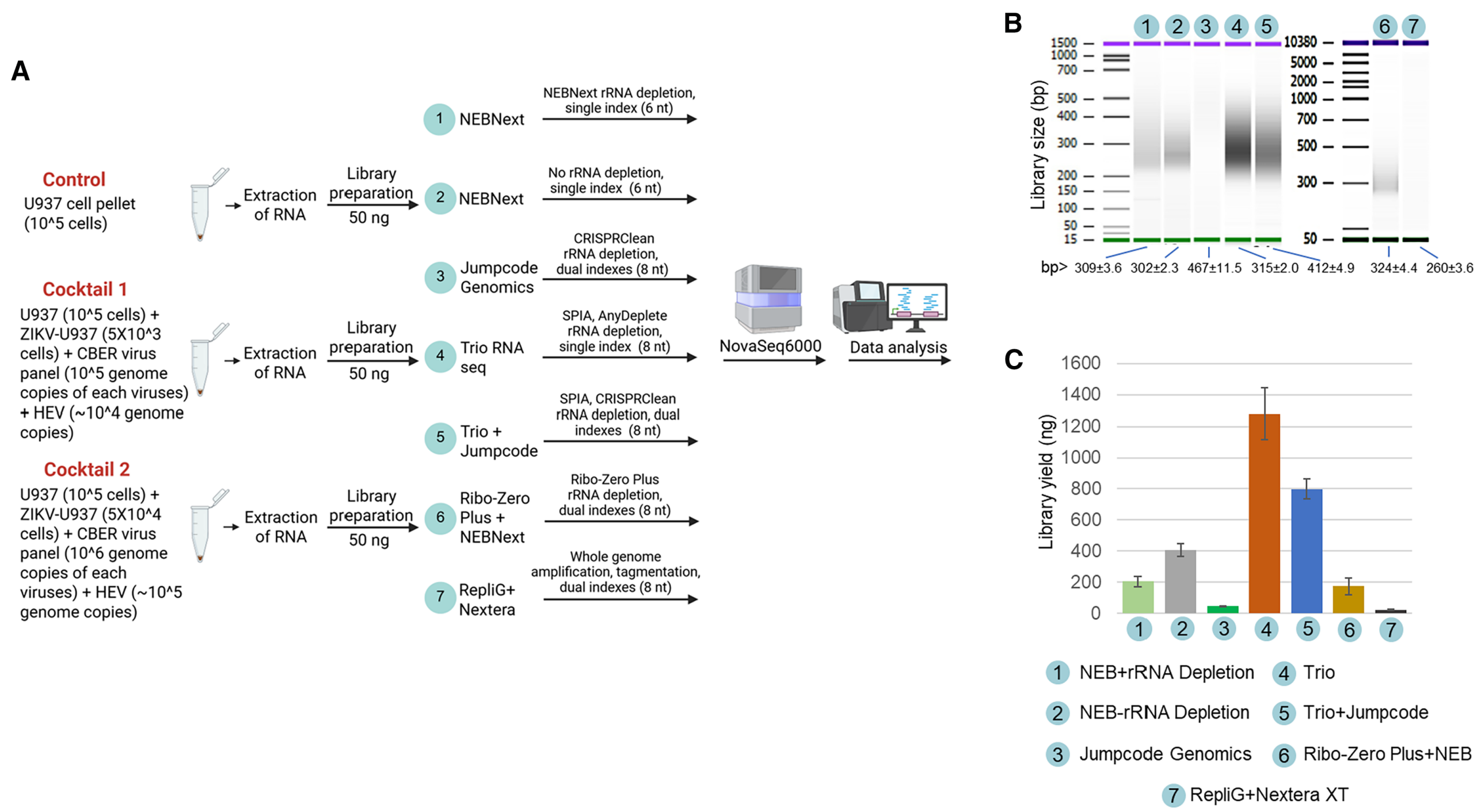

Figure 3. Comparison of Illumina RNA-seq library preparation methods for viral RNA detection

(A) Library preparation protocols with or without rRNA depletion were used to assess viral RNA detection sensitivity. (B) Bioanalyzer traces showing RNA integrity and size distribution of final libraries. The mean NGS library size in bp for each method is shown below, with standard error derived from six libraries generated using each method (control, cocktail 1, and cocktail 2; $n = 2$ biological replicates per condition). (C) Quantification of total library yield by Qubit demonstrates substantial differences across methods; Trio-based protocols yielded the highest amounts. Error bars represent standard error from two independent biological replicates. All library preparations were performed in duplicate ($n = 2$).

were further aligned to the hg38 human genome using HISAT2 (v.2.2.1),[25] and any remaining unmapped reads were interrogated via BLAST for additional taxonomic classification. This initial analysis was performed without host-read pre-filtering and revealed HEV-mapping reads in unspiked control samples (Figure 4B).

To evaluate assay sensitivity, we focused on detecting HEV reads across different sample types—control, cocktail 1, and cocktail 2—prepared using various library preparation kits (Figure 4B). HEV read counts, expressed as counts per million (CPM) along with standard errors, revealed higher HEV signals in unspiked control samples compared to cocktail 2. In some cases, control samples showed HEV read levels comparable to or exceeding those of cocktail 1 (Figure 4B).

To further investigate this HEV signal in controls, we examined the distribution of reads across the HEV C1/p6 Kernow genome (GenBank accession JQ679013) using the Integrative Genomics Viewer (IGV) (Figure 4C).[26] A pronounced read pile-up was observed between positions 2,275–2,446 bp of HEV C1/p6 Kernow genome. This sequence is inserted within the hypervariable region of the HEV C1/p6 Kernow ORF1 protein from the human ribosomal protein S17 (RPS17) gene encoding amino acids 21–76 of RPS17 during cell culturing. This insertion is known to facilitate adaptation of the Kernow C1/p6 HEV strain for replication in cell culture.[19,20]

The apparent inversion in CPM values between unspiked control samples and cocktail 2 reflects a normalization artifact inherent to CPM-based metrics. Low-level HEV-mapping reads in control samples arise from spurious alignment of host-derived transcripts, particularly sequences from the human RPS17 gene, to the HEV reference genome. These background reads remain relatively constant in absolute terms across samples. In contrast, cocktail 2 samples contain substantially higher total mapped reads due to genuine viral sequences from the spiked mixture. Because CPM normalization scales counts relative to total reads, this increased denominator reduces the relative contribution of the fixed background signal, leading to lower apparent HEV CPM values in cocktail 2 compared to controls (Table S2). Based on this finding, we implemented a host-read pre-filtering step for the final viral detection workflow, in which processed reads were first aligned to the human genome and host-unmapped reads were subsequently mapped to the viral reference genomes (Figure 5A).

Evaluation of library preparation methods for viral RNA detection in RNA-seq datasets

RNA-seq analysis was performed on duplicate extractions for control and spiked samples, using each library prep method. Applying the host-filtered mapping workflow, we evaluated the sensitivity of each library prep method for detecting five reference viruses (RSV, FeLV,

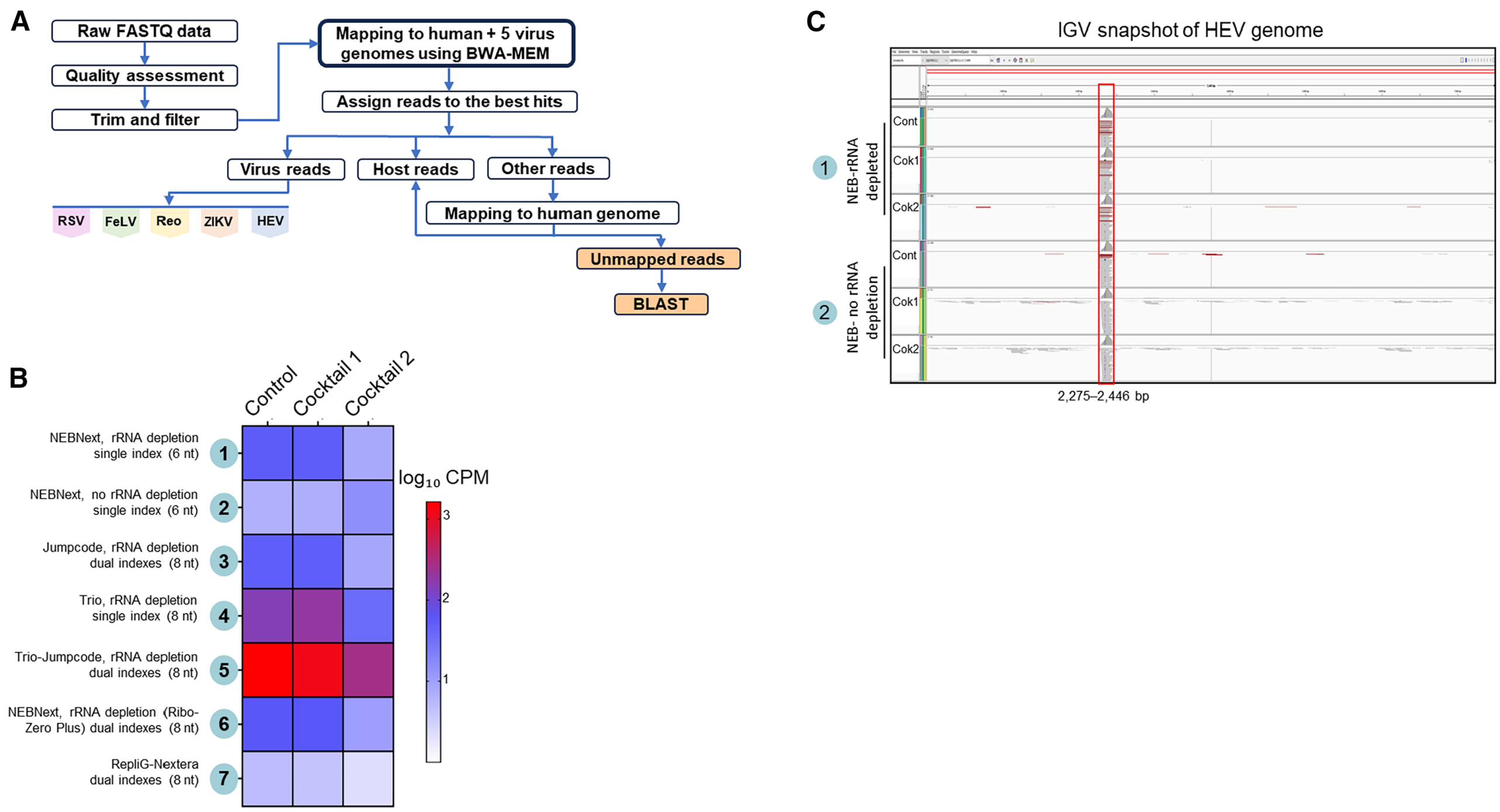

Figure 4. Initial unfiltered RNA-seq mapping reveals host-derived HEV-mapping reads

(A) Initial exploratory bioinformatics pipeline used for mapping transcriptomic reads alignment to human + viral reference genomes, and taxonomic classification of unmapped reads. (B) Mapped read counts (CPM) for HEV across library prep conditions and spike-in groups (control, cocktail 1, and cocktail 2). (C) IGV snapshot showing read accumulation between 2,275 and 2,446 bp on HEV genome due to inserted human RPS17 sequence in the hypervariable region.

orthoreovirus type 1, ZIKV, and HEV) across spiked and control samples (Figures 5A–5C). As five of the seven tested library prep protocols have a ribosomal RNA (rRNA) depletion step integrated, we quantified rRNA content to evaluate the depletion efficiency across library prep methods. As shown in Figure 5B, protocols lacking integrated rRNA depletion-the NEB method (2) and RepliG-Nextera XT (7) exhibited high rRNA levels exceeding 65,000 CPM, indicating no removal of abundant host rRNA. In contrast, all five rRNA depletion protocols worked well, particularly Trio-based protocols (columns 4 and 5) demonstrated significantly lower rRNA content, with counts per million (CPM) consistently below 500 (Table S3).

Impact of library preparation methods on the ability to detect viruses: we then investigated the performance of the library prep methods in amplifying viral RNA. A consistent and notable trend throughout the dataset was the markedly low abundance of viral reads in samples 2 and 7, both of which lacked ribosomal RNA (rRNA) depletion (Figures 5C and S2). These findings underscore the critical role of rRNA depletion in enhancing viral RNA detection sensitivity. A detailed analysis of detection efficiency for each individual virus is presented below (also, see Table S3 for more RNA-seq reads mapping information).

HEV

As anticipated, no HEV reads were detected in most control samples; however, low-level HEV signals were observed in control libraries prepared using the Trio RNA-seq method and the Trio-Jumpcode combination (Figures 5C and S2). The control signal was higher for Trio alone than for Trio-Jumpcode, indicating that both Trio-based workflows can generate low-level background HEV-mapping reads. Among the spiked samples, comparable performance was observed between the NEB kit with rRNA depletion (1), the Jumpcode kit (3), and the Ribo-Zero Plus-NEB kit (6), whereas Trio RNA-seq (4) failed to detect HEV RNA (Figures 5C and S2).

RSV

Trio library preparation methods (4 and 5; Figures 5C and S2) yielded significantly higher numbers of RSV reads compared to all other methods, indicating superior amplification efficiency for RSV RNA. The Jumpcode kit (3) showed the next highest read counts, followed by the Ribo-Zero Plus kit (6) (Figures 5C and S2). Notably, spurious RSV reads were detected in unspiked control libraries prepared using the NEB kits (1 and 2), Trio kit (4), and Trio-Jumpcode kit (5). These reads were absent in control libraries prepared using Jumpcode (3), Ribo-Zero Plus-NEB (6), and RepliG-Nextera XT (7) kits. The presence of false-positive reads in certain control libraries is likely attributable to index hopping. Specifically, methods 3, 5, 6, and 7 used dual 8-nucleotide indexes, whereas methods 1 and 2 used single 6-nucleotide index, and method 4 used single 8-nucleotide index, which are more prone to index misassignment.

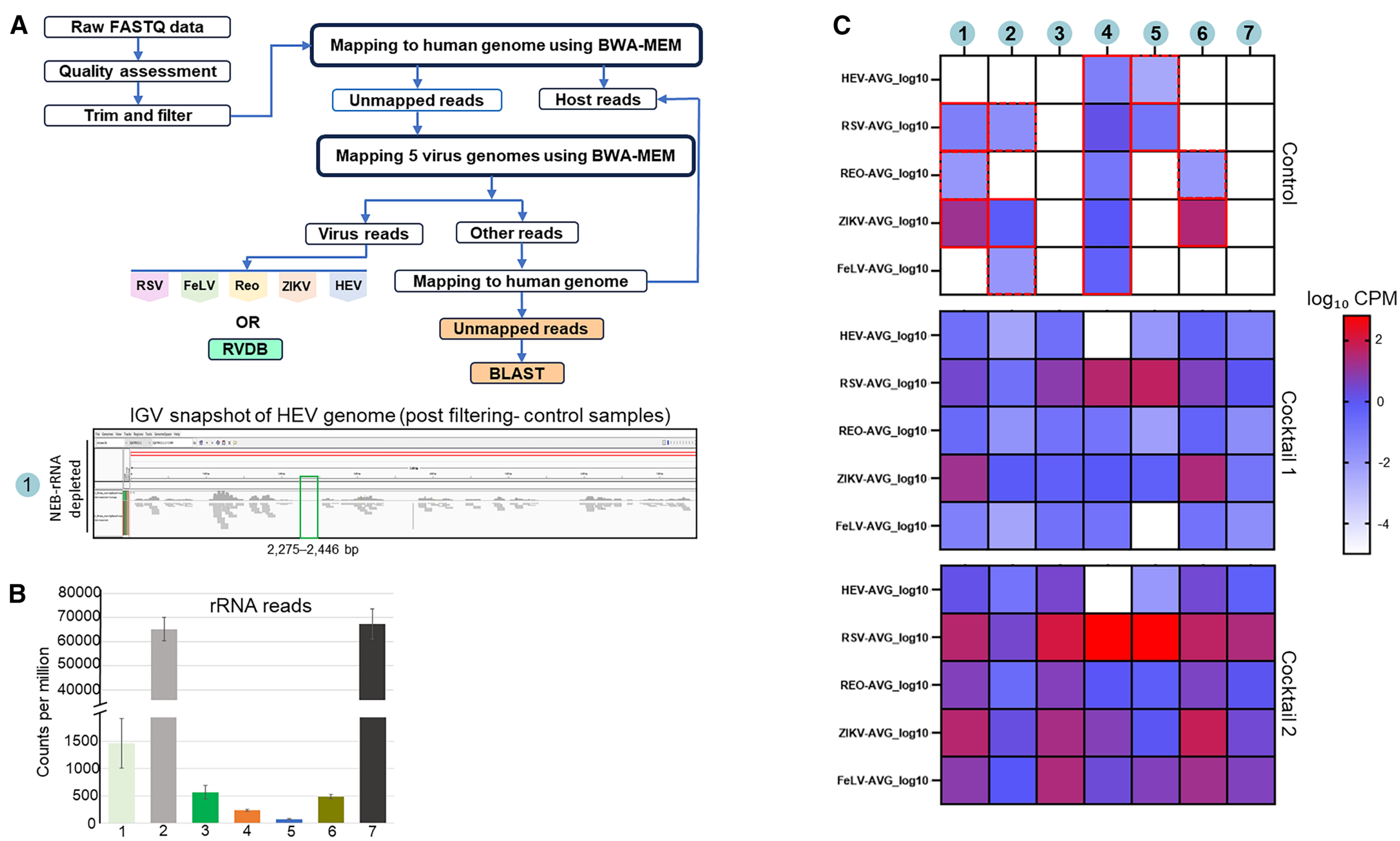

Figure 5. Viral RNA detection efficiency across RNA-seq library prep methods

(A) Upper: final host-filtered bioinformatics pipeline used for host read removal by alignment to human genome followed by mapping to viral reference genomes (host-read pre-filtering before viral mapping) and taxonomic classification of unmapped reads. Lower: IGV snapshot showing no read accumulation between 2,275 and 2,446 bp on HEV genome due to host filtering before mapping to viral genomes. (B) Proportion of reads mapping to ribosomal RNA per method. (C) Heatmaps display $\log_{10}$-transformed CPM values for HEV, RSV, mammalian orthoreovirus 1, ZIKV, and FeLV across library preparation methods and spike-in groups: control (top), cocktail 1 (middle), and cocktail 2 (bottom). Color intensity reflects viral abundance on a $\log_{10}$ CPM scale, ranging from white (not detected) to blue (moderate abundance) and red (high abundance). Corresponding CPM values are provided in Figure S2. Each library preparation method was evaluated using two biological replicates (n = 2). To highlight potential background signal, viruses with detectable reads in control samples are outlined. Solid red borders indicate control signals > 0.05 CPM, whereas dashed red borders indicate trace signals $\leq$ 0.05 CPM.

Mammalian orthoreovirus type 1

For mammalian orthoreovirus type 1, comparable performance was observed across the NEB kit with rRNA depletion (1), the Jumpcode kit (3), and the Ribo-Zero Plus-NEB kit (6) (Figures 5C and S2). Trio and Trio-Jumpcode methods, although were able to detect the mammalian orthoreovirus type 1 in spiked samples, exhibited a lesser performance. Interestingly, we also found this virus reads in non-spiked control samples for the methods NEB, Trio and Ribo-Zero plus, though the number of reads was less compared to spiked samples.

ZIKV

Unexpectedly, we observed significantly elevated levels of ZIKV reads in non-spiked control samples prepared using the NEB (1, 2), Trio alone (4) and Ribo-Zero Plus-NEB kits (6) (Figures 5C, S2, and S3; selected representative examples are shown in Figure S3). In contrast, the Jumpcode kit (3) yielded a substantial number of Zika reads in spiked samples, while no Zika reads were detected in its corresponding control, suggesting high specificity.

To determine whether the elevated ZIKV reads observed in unspiked controls reflected insufficient classification specificity, we re-aligned the ZIKV-mapped reads against an expanded reference panel of closely related flaviviruses (see "methods"). Using paired-end and independent single-end alignment strategies, the reads remained preferentially assigned to ZIKV and did not redistribute to other flaviviruses in the panel (Table S4). These findings indicate that the observed ZIKV signal is unlikely to result from bioinformatics classification errors. However, this analysis does not establish the source of the ZIKV signal, which could reflect reagent-derived contamination or other technical factors, such as cross-contamination.

FeLV

The highest Feline virus reads were observed for Jumpcode kit (3). Ribo-Zero plus-NEB (6) resulted the second highest number of reads (Figures 5C and S2). The lowest number of reads resulted from the NEB without rRNA depletion (2) method. There were reads found

in the control samples for methods NEB without rRNA depletion (2) and Trio (4).

Low-level viral signals were detected in control samples for all the five viruses tested. Attributing these reads solely to index hopping is likely an oversimplification. Rather, these observations are best explained by a combination of factors, including biased PCR amplification, index misassignment, database inaccuracies, crosstalk during multiplexed sequencing, low-level contamination or carryover, and potential alignment or classification artifacts. Collectively, these factors can contribute to false-positive detections in samples expected to be negative. These findings underscore the importance of establishing fit-for-purpose detection thresholds for spiked viruses, as recommended in EP 2.6.41.[15]

Viruses' detection using RVDB

To benchmark the breadth and reproducibility of viral detection across different library preparation conditions, we used Reference Viral DataBase (RVDB) (https://rvdb.dbi.udel.edu/) as the database for our bioinformatic analysis. Table S5 shows all viruses identified using the bioinformatic pipeline shown in Figure 5A (the top 30 viruses are highlighted with a black outline for each individual method). These results represent bioinformatic hits from sequence mapping and were not further qualified as confirmed viral positives. The viral read distribution across methods revealed variation in the ranking of viral species, pointing to differential enrichment or capture efficiencies inherent to each library prep and data analysis methods.

(1) Ubiquitous detection of squirrel monkey retrovirus and human type D retrovirus— similar to earlier reports,[6,27] Squirrel monkey retrovirus was the most abundant virus consistently detected across all conditions and replicates. Its read counts ranged from ∼102.4 to ∼1,639.7 CPM, with the highest observed in method 3. Similarly, human type D retrovirus was detected in all conditions, generally with moderate to high read counts, indicating its strong recovery across multiple library preparations. This consistent overrepresentation suggests that it may be a cell line-derived retrovirus contaminant.

(2) Recurrent detection of spiked viral agents—spiked viruses, such as RSV, FeLV, and mammalian orthoreovirus 1, were recurrently identified in multiple conditions. HRSV was among the top seven most abundant viruses in methods 1 through 7, with CPM ∼588.5 in method 4, highlighting robust detection.

FeLV virus was reliably detected in all conditions, with particularly high read counts in conditions 1, 3, and 6. Mammalian orthoreovirus 1 was also frequently detected but showed prep-dependent dropout from the top 30 viruses list, being undetectable in conditions 5, 6, and 7. These trends highlight the influence of library preparation methodology on the detectability of specific viral taxa.

(3) Library prep-dependent dropout of spiked viral agents—An interesting pattern emerged when comparing the presence or absence of spiked viruses across methods. HEV, for instance, was consistently detected in conditions 1, 2, and 3, but was completely absent from the top 30 in methods 4, 5, 6, and 7. Similarly, mammalian orthoreovirus 1 was present in first four methods but undetectable in the latter three in top 30. This prep-dependent loss of spiked viral agents-particularly mammalian orthoreovirus 1 and HEV-suggests that certain library preparation workflows may not be suitable for comprehensive or agnostic detection of all viral types. Virus-specific physicochemical properties likely contribute to the variability in detection efficiency across library preparation workflows.

(4) Detection of additional viruses—in addition to consistent high-abundance viruses, a wide range of low-to moderate-abundance viruses appeared across conditions. For example, Frijoles virus, rhinovirus A, Rift Valley fever virus, various herpesviruses (e.g., human alphaherpesvirus 1 and 2, gammaherpesvirus 4), and insect- or tick-associated viruses (e.g., Chronaparn virus and Sichuan tick virus) were intermittently detected.

(5) Reproducibility across technical replicates—overall, virus detection patterns were relatively consistent between technical replicates within each condition, with minor variability in read abundance. Most viruses present in one replicate were also found in the other, and their relative rankings were largely preserved, indicating good reproducibility in most preparations. However, a few cases showed moderate replicate variability (e.g., rhinovirus A in condition 4), which may stem from stochastic amplification effects or minor inconsistencies in library construction.

DISCUSSION

NGS is rapidly emerging as a critical tool for adventitious virus detection in biological products due to its unbiased, agnostic nature. However, the analytical performance of NGS-based transcriptomics assays for viral detection depends heavily on upstream steps such as RNA extraction and library preparation, particularly when dealing with complex biological matrices containing low-abundance viral RNA. Our findings demonstrate that both RNA extraction and library preparation strategies exert profound and virus-specific impacts on the sensitivity of viral RNA detection.

Matrix-specific differences in relative viral RNA recovery were evident across the three tested matrices—HEK293, U937, and Buffy Coat. While magnetic bead-based extraction performed best in HEK293 samples, it failed in Buffy Coat due to bead aggregation. Conversely, the QIAGEN method demonstrated the most consistent performance across the different matrices and viral targets evaluated, emerging as the most versatile approach. Notably, the TRI reagent method, despite producing lower total RNA yields in some matrices, showed superior recovery of mammalian orthoreovirus 1 and performed well for several viral targets under specific conditions, highlighting that total RNA yield alone is not necessarily predictive of relative viral RNA recovery. These findings emphasize that extraction method performance is highly dependent on both virus type and sample matrix, and that no single method is universally optimal for all applications. These observations support the ICH Q5A(R2)

guidance, which emphasizes the importance of matrix-specific qualification of nucleic acids extraction methods for NGS-based adventitious virus detection.[4]

Similarly, our data underscore the critical role of rRNA depletion during library preparation specifically regarding pathogen detection. Kits incorporating effective rRNA removal (e.g., Trio, Jumpcode, and Ribo-Zero Plus) yielded lower rRNA content and higher viral read counts compared to those without depletion. The Trio kit, which utilizes SPIA, produced the highest overall library yield and showed superior sensitivity for detecting RSV in spiked samples. However, Trio-based workflows exhibited lower recovery of several other spiked viruses and were also associated with low-level viral signals in negative control samples. These observations raise the possibility that SPIA amplification may introduce amplification biases or background signals that have been reported in highly sensitive sequencing workflows. Notably, low-level viral reads were repeatedly observed in control samples prepared using method 4 and, to a lesser extent, method 2 across multiple viral targets. Although index misassignment likely contributed to some of these signals, particularly given the use of single-indexed libraries, the recurrent detection pattern observed in Trio-based workflows suggests that amplification-related background signal may also contribute. Additional studies designed to independently evaluate amplification chemistry and indexing strategy would be required to distinguish between these potential sources of false-positive reads.

HEV reads in unspiked control libraries warranted further scrutiny. Our IGV-based mapping revealed that the reads aligned to a region of the host-virus recombinant HEV genome, consistent with prior reports describing a 171-nucleotide insertion of RPS17 sequence in the hypervariable region of HEV ORF1 during culture adaptation.[19,20] This finding illustrates how sequence similarity between viral and host genomes can generate false-positive signals, especially when host reads are not adequately filtered. To mitigate such issues, we implemented a prefiltering pipeline that removed host reads prior to viral genome mapping, an essential step to improve specificity in pathogen detection workflows.

Moreover, our side-by-side comparison revealed that indexing strategy plays a substantial role in minimizing cross-contamination when multiplexing during sequencing. Kits using dual 8-nucleotide indexes (e.g., RepliG-Nextera XT and Jumpcode) exhibited no or reduced index misassignment compared to those using single 6- or 8-nucleotide indexes, a known contributor to spurious viral detection in high-sensitivity NGS datasets.[28] Also, the use of non-patterned flow cells (Illumina), unique molecular identifiers (UMIs), and optimized library loading can help mitigate these contamination issues.

One noteworthy finding of this study was the detection of viral sequences at low to moderate abundance in control samples when using an untargeted bioinformatics pipeline. An exception was observed with the Jumpcode method, which incorporates rRNA depletion after library construction; when combined with dual indexing of eight nucleotides, this approach markedly improved viral signal detection, suggesting that rRNA depletion performed post-PCR amplification may be more effective. In contrast, viral RNA detections observed in other control samples are likely attributable to non-specific PCR amplification, bioinformatic artifacts, including misalignment of NGS reads to unrelated taxa, spurious alignments to low-complexity or repetitive genomic regions, or inaccuracies within reference viral genome sequences in the databases used for analysis. Additionally, the possibility of environmental contamination or contamination introduced through nucleic acid extraction and library preparation reagents cannot be excluded. When using untargeted bioinformatics pipelines for viral detection, it is crucial to establish acceptable thresholds for defining a positive hit and to implement a robust follow-up strategy to mitigate these issues.[15]

Our study provides empirical evidence supporting a risk-based approach to NGS method development and validation. Critical assay components-such as extraction reagents, library kits, and informatics filters-must be qualified not in isolation, but in the context of specific product matrices and potential viral contaminants. The reference ZIKV-infected U937 cell line developed here offers a biologically relevant benchmark for future assay optimization and qualification, particularly for flavivirus detection.

MATERIAL AND METHODS

Viral stocks and dilutions

Both BEI NR-59622[29] and ATCC MSA 2008 virus panels were used for spiking different matrices for the evaluation of different RNA extraction methods. BEI NR-59622 was used for the evaluation of different library preparation methods. BEI NR-59622, CBER virus panel contains five viruses, including mammalian orthoreovirus 1, feline leukemia virus (FeLV), respiratory syncytial virus (RSV), porcine circovirus (PCV), and Epstein-Barr virus (EBV), were serially diluted in 10-fold using sterile phosphate buffered saline (PBS) from 10^{-1} to 10^{-7}. Few aliquots of each dilution were stored at -80° for further use. HEV RR is a lyophilized reference reagent prepared from heat-inactivated, cell culture propagated molecular clone of HEV (Kernow C1 p6; GenBank accession number JQ679013.1).[23] HEV RR and persistently ZIKV-infected U937 cells were also used for spiking experiments.

Generation of persistent ZIKV infected U937 cells

To generate a cell line persistently infected with ZIKV, U937 cells ($\sim 1 \times 10^{6}$) were infected with the ZIKV MR766 strain at a multiplicity of infection (MOI) of 1. Infections were carried out in culture medium supplemented with 2% fetal bovine serum (FBS) and penicillin-streptomycin (P/S). After a 2-h adsorption, the viral inoculum was removed, and cells were maintained in complete growth medium containing 10% FBS and P/S. The infected cultures were subsequently passaged on a weekly basis for a minimum of 3 months. The immunofluorescence assay was performed to confirm persistently infected ZIKV cells compared to uninfected control cells using anti-4G2 flavivirus antibody.

BEI NR-59622 spiked into U937 for library preparation

A control U937 cell pellet (10^5 cells) was resuspended in 251 μL of PBS to achieve the required starting volume for the extraction method. To generate the test samples, a naive U937 cell pellet (10^5 cells) was spiked with the viral panel from BEI NR-59622 and mixed thoroughly to create cocktail 1 and cocktail 2 (Figure 3A). In addition, persistently infected U937-ZIKV cells and HEV reference reagent (HEV RR) were added to both cocktails. For the ZIKV component, 50 μL of persistently infected U937-ZIKV cells at concentrations of 10^5 cells/mL and 10^6 cells/mL were added to cocktail 1 and cocktail 2, respectively. Similarly, 41 μL of HEV RR containing 2.4×10^5 genome copies/mL and 2.4×10^6 genome copies/mL were added to cocktail 1 and cocktail 2, respectively. The total viral spike volume for each cocktail was 251 μL. Samples were extracted in duplicate using the TRI Reagent-PureLink method. To eliminate residual DNA contamination, on-column DNase treatment was performed during RNA extraction using PureLink DNase (Invitrogen, Cat. No. 12185010). No carrier RNA was used during extraction of samples used for the RNA-seq library preparation experiments.

Spiking different matrices for evaluation of relative viral RNA recovery

Reference viral panel ATCC MSA 2008 spiked into HEK293 or U937

100 μL of virome mix MSA 2008 was spiked into HEK293 or U937 (10^5 cells) cell pellet at 1:1 ratio (genome copy of viruses:cells) and briefly vortexed to mix. The samples were then extracted in duplicate using different RNA extraction methods and evaluated.

BEI NR-59622 virus panel spiked into HEK293

Mammalian orthoreo virus 1, FeLV, RSV viruses from BEI-NR-59622 were spiked into HEK293 cell pellet (10^5 cells) at 1:1 ratio (genome copy of viruses:cells) and briefly vortexed to mix. Besides those viruses, 50 μL of 10^6 Zika persistently infected U937 cells/mL and 41 μL of 4×10^5 genome copies/mL HEV RR were spiked into the cell pellet. Total spiking volume of the viruses was 191 μL. The samples were then extracted in duplicate using different RNA extraction methods and evaluated.

Note that excessive vortexing can potentially disrupt viral particles, particularly for enveloped viruses, and may affect infectivity or structural integrity. However, in this study the viruses were used for nucleic acid-based detection (qRT-PCR and RNA-seq) rather than infectivity assays, and the brief vortexing step was applied only to ensure homogeneous mixing of the spike-in material. Because viral RNA is the analyte of interest, and lysis is subsequently performed during extraction, we do not expect this step to significantly impact RNA recovery or detection. Nevertheless, we acknowledge that mechanical stress could theoretically influence RNA recovery for certain virus types.

Reference viral panel ATCC MSA 2008 spiked into buffy coat

A 100 μL aliquot of the ATCC MSA-2008 reference viral panel, containing 10^5 genome copies, was spiked into 140 μL of buffy coat and mixed thoroughly prior to nucleic acid extraction.

RNA extraction methods

To evaluate relative viral RNA recovery across the different RNA extraction methods, RNA exactions were carried out using different extraction kits/methods, 1–5: TRI Reagent (Zymo research, R2050-1-200) chloroform, PureLink RNA Mini kit (Invitrogen, 12183025), TRI Reagent plus PureLink RNA Mini kit, QIAGEN Viral RNA Mini kit (QIAGEN, 52906), and RNA extraction with Magnetic Beads RNAClean XP (Beckman Coulter, A63987) following manufacturer instructions. The cells and viruses were thawed before use, and cells were centrifuged at 500 × *g* for 3 min before spiking the viruses. The control cell was combined with PBS to obtain the appropriate starting volume and used for RNA extraction. For the QIAGEN Viral RNA Mini Kit, carrier RNA was added to Buffer AVL according to the extraction protocol. Therefore, the Qubit measurement of RNA extracted using the QIAGEN method includes both sample-derived RNA and carrier RNA. Consequently, Qubit-based total RNA yield for the QIAGEN method should not be directly compared with yields from extraction methods in which carrier RNA was not used. For the magnetic bead extraction method, 300 μL of lysis buffer from PureLink RNA Mini kit containing 1% beta marcaptoethanol was added to the sample and mixed vigorously by vortexing. Later, 1.8 times RNAClean XP beads was added to the lysed reaction volume and followed the RNAClean XP protocol to extract the RNA. Following manufacturer's instructions, carrier RNA was added to the QIAGEN lysis buffer AVL. For PureLink RNA Mini kit, TRI Reagent plus PureLink RNA Mini kit, QIAGEN Viral RNA Mini kit (QIAGEN, 52906) on column DNase treatment was performed during RNA extraction. In tube DNase (Sigma-Aldrich, AMPD1-1KT) treatment was done for Tri Reagent-chloroform and Magnetic beads methods. Because in-tube DNase treatment required the addition of 20 μL of reagents to RNA extracted using the TRI Reagent-chloroform and magnetic bead methods, nuclease-free water was added to RNA samples extracted using the PureLink RNA Mini Kit, TRI Reagent plus PureLink RNA Mini Kit, and QIAGEN Viral RNA Mini Kit to maintain equivalent sample volumes across all extraction methods.

After extraction, the total RNA quantity was measured using the Qubit 4 fluorometer (Thermo Fisher Scientific). The extracted RNA of viruses was detected using quantitative real-time PCR. Although the QIAGEN Viral RNA Mini Kit is intended for cell-free samples, it was used here with small cell pellets ($\leq 10^6$ cells), which lysed efficiently without column clogging. In contrast, more complex matrices (e.g., buffy coat) exhibited reduced lysis efficiency and required longer processing times, a pattern also observed with the Invitrogen PureLink method. Despite being designed for cell-free applications, the chaotropic chemistry of AVL from QIAGEN Viral RNA Mini Kit supported effective lysis and RNase inactivation under these conditions, enabling direct comparison with the PureLink method, which includes guanidinium salt and β-mercaptoethanol effective lysis and RNase inactivation.

qRT-PCR

All qRT-PCR tests were performed using a Bio-Rad CFX96 Touch Real-Time PCR Detection System using Bio-Rad iTaq Universal

SYBR Green One-Step Kit (Bio-Rad, 1725151) containing 2× master mix, reverse transcriptase, and nuclease-free water. The PCR reaction was performed in 20 μL reaction volume containing 10 μL of 2× mastermix, 1 μL of 5 μM forward and reverse primers which gives the final primer concentration 0.25 μM in the reaction, 0.25 μL of reverse transcriptase, 3.5 μL of RNA, and adjusted rest of the volume with water. The primers sequences for RSV, FeLV and Orthoreo virus were provided by Dr. Arifa Khan. Primer sequences for ZIKV and HEV are provided elsewhere.[30,31] Thermocycling conditions were: 50°C for 10 min, 95°C 2 min, followed by 40 cycles of: 95°C 15 s, 57°C plate read, followed by a melt curve from 65°C to 95°C. See Table S1 for CT values.

RT-qPCR CT values were used as a relative measure of viral RNA recovery. CT values were compared only among extraction methods for the same virus within the same sample matrix. No standard curves were generated for absolute quantification, and amplification efficiencies were not calculated. Therefore, CT values should be interpreted as comparative indicators of extraction performance rather than absolute viral genome copy numbers or extraction efficiency.

Library preparation methods

For the preparation of the library, seven library preparation kits were used according to manufacturer instructions or slight modifications. The kits used for the study were (1) NEBNext rRNA Depletion Kit (E7400L) with NEBNext Ultra II Directional RNA Library Prep Kit for Illumina (E7760L), (2) NEBNext Ultra II Directional RNA Library Prep Kit for Illumina (E7760L), (3) Jumpcode Genomics kit (KIT1014), (4) Trio RNA-seq kit, (5) Trio RNA-seq kit+ Jumpcode Genomics kit (KIT1014), (6) Illumina Ribo-Zero Plus rRNA Depletion Kit (20040526)+NEBNext Ultra II Directional RNA Library Prep Kit for Illumina (E7760L) with dual 8 nucleotide indexes from NEBNext Multiplex Oligos for Illumina, and (7) REPLI-g Single Cell kit (QIAGEN, 150343) + Nextera XT DNA Library Preparation kit (FC-131-1024) (see Figure 3). Duplicate RNA-seq libraries were prepared from 50 ng of DNase treated total RNA using different library preparation kits following manufacturer instructions or some modifications such as Trio+Jumpcode method and RepliG+Nextera method where hybrid protocol was used. For combining Trio+ Jumpcode method (5), we performed a hybrid library preparation by using Trio RNA-seq for synthesizing cDNA and SPIA amplification and Jumpcode Genomics Kit to construct the sequencing library (Illumina, San Diego, CA, USA), using manufacturer protocol. For RepliG+Nextera method, first strand cDNA was synthesized from DNase treated RNA using SuperScript III First-Strand Synthesis SuperMix (Invitrogen, 18080400). Next, whole genome was amplified using REPLI-g Single Cell kit. The amplified DNA then used to construct library preparation using Nextera XT DNA kit. The prepared libraries were analyzed on Bioanalyzer to determine fragment length before sequencing the libraries. The libraries were also quantified using a Qubit 4 fluorometer. All extractions and library preparations were performed in duplicate ($n = 2$).

Sequencing

The libraries were sequenced using Illumina NovaSeq 6000 S1 Reagent Kit v1.5 (300 cycles, 20028317) and NovaSeq 6000 SP Reagent Kit v1.5 (200 cycles, 20040719) in NovaSeq 6000 S1 and SP flow cells. See Table S6 for batchability information.

Bioinformatic data analysis

Sources of sequences

- Human genomic sequences
  - hg38
    - https://s3.amazonaws.com/igenomes.illumina.com/Homo_sapiens/NCBI/GRCh38/Homo_sapiens_NCBI_GRCh38.tar.gz
- Virus sequences
  - Feline leukemia virus (FeLV) (Thielen strain)-FeLV
    - Taxon: Feline leukemia virus
    - Strain: KT-FeLV-UCD-1
    - Complete genome (GenBank: MT129531.1)
    - May 4, 2020
    - https://www.ncbi.nlm.nih.gov/nuccore/MT129531.1
  - Human respiratory syncytial virus (RSV) subtype A (A2 strain)-RSV. The RSV present in the BEI NR-59622 spike-in panel was strain A2. For bioinformatic read mapping, the RSV reference sequence A/WI/629-3/06-07 was used.
    - Taxon: Human respiratory syncytial virus
    - Strain: A/WI/629-3/06-07
    - Complete genome (GenBank: JF920069.1)
    - September 12, 2014
    - https://www.ncbi.nlm.nih.gov/nuccore/JF920069.1
  - Human reovirus type 1 (REO1) (Lang strain)-Reo
    - Taxon: Mammalian orthoreovirus 1 Lang
    - M24734.1 (L1), AF378003.1 (L2), AF129820.1 (L3), AF461682.1 (M1), AF490617.1 (M2), AF174382.1 (M3), M10260.1 (S1), L19774.1 (S2), M18389.1 (S3), X61586.1 (S4)
  - ZIKV
    - Taxon: Zika virus
    - Strain: MR 766
    - Complete genome (GenBank: AY632535.2, RefSeq: NC_012532.1)
    - https://www.ncbi.nlm.nih.gov/nuccore/NC_012532.1
  - HEV
    - Taxon: Hepatitis E virus clone p6
    - Strain: Kernow C1 p6
    - Complete genome (GenBank: JQ679013.1)
    - April 30, 2012
    - https://www.ncbi.nlm.nih.gov/nuccore/JQ679013

Combined genomes

Five viruses

The complete or partial genome sequences from the five viruses (downloaded from the links above) were pooled together to make a combined genome named hg38 + 5 virus genomes.

RVDB

The clustered (C-) nucleotide sequences were downloaded from Reference Viral DataBase (RVDB, version 29.0, August 8, 2024). The sequences from RVDB were pooled together to make a combined genome named RVDBv29.

Sequencing data processing and mapping to combined genomes

Raw FASTQ data quality was assessed using FastQC 0.12.1 (https://www.bioinformatics.babraham.ac.uk/projects/fastqc/) and MultiQC 1.14[32] with default parameters. Following quality assessment, which revealed high data quality, adapter sequences, and 3′ end polymers were removed using fastp 0.23.4.[33]

Initial exploratory mapping analysis—for the initial analysis shown in Figure 4A, processed reads were aligned against a combined reference containing the human genome and five viral genomes (RSV, FeLV, mammalian orthoreovirus type 1, ZIKV, and HEV) using BWA-MEM. Reads remaining unmapped after this analysis were further aligned to hg38 using HISAT2, and the remaining unmapped reads were analyzed by BLAST for additional taxonomic classification. This exploratory analysis was performed without host-read pre-filtering which identified HEV-mapping signal in control samples.

Final host-filtered viral mapping workflow—processed read pairs were aligned to the human reference genome (hg38) using BWA-MEM 0.7.19[24] with default parameters. Read counts for rRNA genes were quantified using featureCounts 2.1.1[34] based on the ncbiRefSeq gene model, with counting restricted to read pairs where both ends mapped to hg38. Both the BWA-MEM genome index and ncbiRefSeq gene model were obtained from Illumina iGenomes (https://support.illumina.com/sequencing/sequencing_software/igenome.html). Read pairs where both ends were unmapped to hg38 were classified as unmapped-to-host and extracted for subsequent viral analysis.

Unmapped-to-host read pairs were aligned to the two combined viral reference genomes, five viruses and RVDBv29, using BWA-MEM with default parameters. Mapped read pairs were quantified for each virus, with counting limited to read pairs where both ends mapped to the same viral genome.

For untargeted RVDB-based analysis, only primary mapped read pairs were retained. For viruses represented by multiple contigs or genome segments, a read pair was counted only when both reads mapped to the same annotated virus. Read counts were then summarized at the virus level according to RVDB annotation. These assignments were interpreted as screening-level bioinformatic hits arising from low-complexity reads, homologous regions, and database limitations and were not considered confirmed viral detections without additional confirmatory evaluation.

Competitive flavivirus re-alignment of ZIKV-mapped reads

To assess whether the unexpected reads mapping to ZIKV in control samples could result from misalignment of bioinformatic analysis, reads previously assigned to ZIKV were extracted and re-aligned against an expanded competitive flavivirus reference panel. The panel comprised ZIKV strain MR 766 (NC_012532.1), Spondweni virus strain SM-6 V-1 (DQ859064.1), dengue virus serotypes 1–4 (NC_001477.1, NC_001474.2, NC_001475.2, and NC_002640.1, respectively), Ilheus virus (NC_009028.2), Rocio virus strain SPH 34675 (NC_040776.1), Saint Louis encephalitis virus (NC_007580.2), West Nile virus lineage 1 (NC_009942.1), Japanese encephalitis virus (NC_001437.1), and yellow fever virus (NC_002031.1).

Competitive re-alignment was performed independently using BWA-MEM. Two mapping strategies were evaluated: (1) paired-end alignment, in which R1 and R2 were aligned jointly, and (2) independent single-end alignment, in which R1 and R2 were aligned separately, and the resulting alignments were subsequently combined and evaluated by read-pair identifier. Primary and alternative alignments were examined using the alignment score (AS) and mapping quality (MAPQ) reported by the aligner to assess whether individual reads preferentially aligned to ZIKV or to another flavivirus in the expanded reference panel.

Human ribosomal RNA genes (NCBI RefSeq)

18S: RNA18SN1, RNA18SN2, RNA18SN3, RNA18SN4, RNA18SN5.

18S ribosomal pseudogene: RNA18SP, RNA18SP2, RNA18SP3, RNA18SP4, RNA18SP5.

28S: RNA28SN1, RNA28SN2, RNA28SN3, RNA28SN4, RNA28SN5.

5.8S: RNA5-8SN1, RNA5-8SN2, RNA5-8SN3, RNA5-8SN4, RNA5-8SN5.

5.8S ribosomal pseudogene: RNA5-8SP, RNA5-8SP2, RNA5-8SP3, RNA5-8SP4, RNA5-8SP5, RNA5-8SP6, RNA5-8SP7, RNA5-8SP8, RNA5-8SP10.

5S: RNA5S1, RNA5S2, RNA5S3, RNA5S4, RNA5S5, RNA5S6, RNA5S7, RNA5S8, RNA5S9, RNA5S10, RNA5S11, RNA5S12, RNA5S13, RNA5S14, RNA5S15, RNA5S16, RNA5S17.

5S ribosomal pseudogene: RNA5SP18, RNA5SP19, RNA5SP20, RNA5SP21, RNA5SP22, RNA5SP23, RNA5SP24, RNA5SP25, RNA5SP26, RNA5SP27, RNA5SP28, RNA5SP29, RNA5SP30, RNA5SP31, RNA5SP33, RNA5SP34, RNA5SP35, RNA5SP36, RNA5SP37, RNA5SP38, RNA5SP39, RNA5SP40, RNA5SP41, RNA5SP42, RNA5SP43, RNA5SP44, RNA5SP45, RNA5SP46, RNA5SP47, RNA5SP48, RNA5SP49, RNA5SP50, RNA5SP51, RNA5SP52, RNA5SP53, RNA5SP54, RNA5SP55, RNA5SP56, RNA5SP57, RNA5SP59, RNA5SP60, RNA5SP61, RNA5SP62,

RNA5SP63, RNA5SP64, RNA5SP65, RNA5SP66, RNA5SP67, RNA5SP68, RNA5SP69, RNA5SP70, RNA5SP71, RNA5SP72, RNA5SP73, RNA5SP74, RNA5SP75, RNA5SP76, RNA5SP77, RNA5SP78, RNA5SP79, RNA5SP80, RNA5SP82, RNA5SP84, RNA5SP85, RNA5SP86, RNA5SP87, RNA5SP88, RNA5SP89, RNA5SP90, RNA5SP91, RNA5SP92, RNA5SP93, RNA5SP94, RNA5SP95, RNA5SP96, RNA5SP97, RNA5SP98, RNA5SP99, RNA5SP100, RNA5SP101, RNA5SP102, RNA5SP103, RNA5SP104, RNA5SP105, RNA5SP106, RNA5SP107, RNA5SP108, RNA5SP109, RNA5SP110, RNA5SP111, RNA5SP112, RNA5SP113, RNA5SP114, RNA5SP115, RNA5SP116, RNA5SP117, RNA5SP118, RNA5SP119, RNA5SP120, RNA5SP121, RNA5SP122, RNA5SP123, RNA5SP124, RNA5SP125, RNA5SP126, RNA5SP127, RNA5SP128, RNA5SP129, RNA5SP130, RNA5SP131, RNA5SP132, RNA5SP133, RNA5SP134, RNA5SP135, RNA5SP136, RNA5SP137, RNA5SP138, RNA5SP139, RNA5SP140, RNA5SP141, RNA5SP142, RNA5SP143, RNA5SP144, RNA5SP145, RNA5SP146, RNA5SP147, RNA5SP148, RNA5SP149, RNA5SP150, RNA5SP151, RNA5SP152, RNA5SP153, RNA5SP154, RNA5SP155, RNA5SP156, RNA5SP157, RNA5SP158, RNA5SP159, RNA5SP160, RNA5SP161, RNA5SP162, RNA5SP163, RNA5SP164, RNA5SP165, RNA5SP166, RNA5SP167, RNA5SP168, RNA5SP169, RNA5SP170, RNA5SP171, RNA5SP172, RNA5SP173, RNA5SP174, RNA5SP175, RNA5SP176, RNA5SP177, RNA5SP178, RNA5SP179, RNA5SP180, RNA5SP181, RNA5SP182, RNA5SP183, RNA5SP184, RNA5SP185, RNA5SP186, RNA5SP187, RNA5SP188, RNA5SP189, RNA5SP190, RNA5SP191, RNA5SP192, RNA5SP193, RNA5SP194, RNA5SP195, RNA5SP196, RNA5SP197, RNA5SP198, RNA5SP199, RNA5SP200, RNA5SP201, RNA5SP202, RNA5SP203, RNA5SP204, RNA5SP205, RNA5SP206, RNA5SP207, RNA5SP208, RNA5SP209, RNA5SP210, RNA5SP211, RNA5SP212, RNA5SP213, RNA5SP214, RNA5SP215, RNA5SP216, RNA5SP217, RNA5SP218, RNA5SP219, RNA5SP220, RNA5SP221, RNA5SP222, RNA5SP223, RNA5SP224, RNA5SP225, RNA5SP226, RNA5SP227, RNA5SP228, RNA5SP229, RNA5SP230, RNA5SP231, RNA5SP232, RNA5SP233, RNA5SP234, RNA5SP235, RNA5SP236, RNA5SP237, RNA5SP238, RNA5SP239, RNA5SP240, RNA5SP241, RNA5SP242, RNA5SP243, RNA5SP244, RNA5SP245, RNA5SP246, RNA5SP247, RNA5SP248, RNA5SP249, RNA5SP250, RNA5SP251, RNA5SP252, RNA5SP253, RNA5SP254, RNA5SP255, RNA5SP256, RNA5SP257, RNA5SP258, RNA5SP259, RNA5SP260, RNA5SP261, RNA5SP262, RNA5SP263, RNA5SP264, RNA5SP265, RNA5SP266, RNA5SP267, RNA5SP268, RNA5SP269, RNA5SP270, RNA5SP271, RNA5SP272, RNA5SP273, RNA5SP274, RNA5SP275, RNA5SP276, RNA5SP277, RNA5SP278, RNA5SP279, RNA5SP280, RNA5SP281, RNA5SP282, RNA5SP283, RNA5SP284, RNA5SP285, RNA5SP286, RNA5SP287, RNA5SP288, RNA5SP289, RNA5SP290, RNA5SP291, RNA5SP292, RNA5SP293, RNA5SP294, RNA5SP295, RNA5SP296, RNA5SP297, RNA5SP298, RNA5SP299, RNA5SP300, RNA5SP301, RNA5SP302, RNA5SP303, RNA5SP304, RNA5SP305, RNA5SP306, RNA5SP307, RNA5SP308, RNA5SP309, RNA5SP310, RNA5SP311, RNA5SP312, RNA5SP315, RNA5SP317, RNA5SP318, RNA5SP319, RNA5SP320, RNA5SP321, RNA5SP322, RNA5SP323, RNA5SP324, RNA5SP325, RNA5SP326, RNA5SP327, RNA5SP328, RNA5SP329, RNA5SP330, RNA5SP331, RNA5SP332, RNA5SP333, RNA5SP334, RNA5SP335, RNA5SP336, RNA5SP337, RNA5SP338, RNA5SP339, RNA5SP340, RNA5SP341, RNA5SP342, RNA5SP343, RNA5SP344, RNA5SP345, RNA5SP346, RNA5SP347, RNA5SP348, RNA5SP349, RNA5SP350, RNA5SP351, RNA5SP352, RNA5SP353, RNA5SP354, RNA5SP355, RNA5SP356, RNA5SP357, RNA5SP358, RNA5SP359, RNA5SP360, RNA5SP361, RNA5SP362, RNA5SP363, RNA5SP364, RNA5SP365, RNA5SP366, RNA5SP367, RNA5SP368, RNA5SP369, RNA5SP370, RNA5SP371, RNA5SP372, RNA5SP373, RNA5SP374, RNA5SP375, RNA5SP376, RNA5SP377, RNA5SP378, RNA5SP379, RNA5SP380, RNA5SP382, RNA5SP383, RNA5SP384, RNA5SP385, RNA5SP386, RNA5SP387, RNA5SP388, RNA5SP389, RNA5SP390, RNA5SP391, RNA5SP392, RNA5SP393, RNA5SP394, RNA5SP395, RNA5SP396, RNA5SP397, RNA5SP399, RNA5SP400, RNA5SP401, RNA5SP402, RNA5SP403, RNA5SP404, RNA5SP405, RNA5SP406, RNA5SP407, RNA5SP408, RNA5SP409, RNA5SP410, RNA5SP411, RNA5SP412, RNA5SP413, RNA5SP414, RNA5SP415, RNA5SP416, RNA5SP417, RNA5SP418, RNA5SP419, RNA5SP420, RNA5SP421, RNA5SP422, RNA5SP423, RNA5SP424, RNA5SP425, RNA5SP426, RNA5SP427, RNA5SP428, RNA5SP429, RNA5SP430, RNA5SP431, RNA5SP432, RNA5SP433, RNA5SP434, RNA5SP435, RNA5SP436, RNA5SP437, RNA5SP438, RNA5SP439, RNA5SP440, RNA5SP441, RNA5SP442, RNA5SP443, RNA5SP444, RNA5SP445, RNA5SP446, RNA5SP447, RNA5SP448, RNA5SP449, RNA5SP450, RNA5SP451, RNA5SP452, RNA5SP453, RNA5SP454, RNA5SP455, RNA5SP456, RNA5SP457, RNA5SP458, RNA5SP459, RNA5SP460, RNA5SP461, RNA5SP462, RNA5SP463, RNA5SP464, RNA5SP465, RNA5SP466, RNA5SP467, RNA5SP468, RNA5SP469, RNA5SP470, RNA5SP471, RNA5SP472, RNA5SP473, RNA5SP474, RNA5SP475, RNA5SP476, RNA5SP477, RNA5SP478, RNA5SP479, RNA5SP480, RNA5SP481, RNA5SP482, RNA5SP483, RNA5SP484, RNA5SP485, RNA5SP486, RNA5SP487, RNA5SP488, RNA5SP489, RNA5SP490, RNA5SP491, RNA5SP492, RNA5SP493, RNA5SP494, RNA5SP495, RNA5SP496, RNA5SP497, RNA5SP498, RNA5SP499, RNA5SP500, RNA5SP501, RNA5SP502, RNA5SP503, RNA5SP504, RNA5SP505, RNA5SP506, RNA5SP507, RNA5SP508, RNA5SP509, RNA5SP510, RNA5SP511, RNA5SP512, RNA5SP513, RNA5SP514, RNA5SP515, RNA5SP516, RNA5SP517, RNA5SP518, RNA5SP519, RNA5SP520, RNA5SP521, RNA5SP522, RNA5SP523, RNA5SP524, RNA5SP525, RNA5SP526, RNA5SP527, RNA5SP528, RNA5SP529, RNA5SP530, RNA5SP531, RNA5SP532, RNA5SP533, RNA5SP534, RNA5SP535, RNA5SP536.

DATA AND CODE AVAILABILITY

All data needed to evaluate the conclusions in the paper are present in the paper and/or the supplemental materials. RNA-seq data can be found here: https://dataview.ncbi.nlm.nih.gov/object/PRJNA1478660?reviewer=gm5vo2frf9bucdc4b6167a7ens.

ACKNOWLEDGMENTS

The authors thank Dr. David McGivern for providing HEV secondary standards. We thank Dr. Arifa Khan for providing the primers sequences for RSV, FeLV, and Orthoreo virus. We also thank Drs. David McGivern, Fei Mo, and Bharat Joshi for critical reading of this manuscript and helpful comments. This work was supported by the Intramural Research Program of the Center for Biologics Evaluation and Research (CBER), US Food and Drug Administration. This project was also supported in part by Sayma Afroj and Aaron Scholl's appointment to the Research Participation Program at CBER administered by the Oak Ridge Institute for Science and Education through the US Department of Energy and US Food and Drug Administration. Ethics approval and consent to

participate—not applicable. The graphical abstract was created using BioRender (https://BioRender.com).

AUTHOR CONTRIBUTIONS

S.A., A.S., B.L., and S.D. contributed to the conceptualization of the study; methodology was developed by S.A., B.G., A.S., B.L., J.X., and S.D.; S.A., B.G., J.X., and S.D. conducted the investigation; B.G. and S.D. were responsible for visualization; J.X. and S.D. provided supervision; the original draft of the manuscript was prepared by S.A., B.G., and S.D. All authors (S.A., B.G., A.S., B.L., J.X., and S.D.) contributed to the review and editing of the manuscript and approved the final version.

DECLARATION OF INTERESTS

The authors declare no competing interests.

SUPPLEMENTAL INFORMATION

Supplemental information can be found online at https://doi.org/10.1016/j.omta.2026.201858.

REFERENCES

1. Gu, W., Deng, X., Lee, M., Sucu, Y.D., Arevalo, S., Stryke, D., Federman, S., Gopez, A., Reyes, K., Zorn, K., et al. (2021). Rapid pathogen detection by metagenomic next-generation sequencing of infected body fluids. Nat. Med. *27*, 115–124. https://doi.org/10.1038/s41591-020-1105-z.
2. Armstrong, A.E., Rossoff, J., Hollemon, D., Hong, D.K., Muller, W.J., and Chaudhury, S. (2019). Cell-free DNA next-generation sequencing successfully detects infectious pathogens in pediatric oncology and hematopoietic stem cell transplant patients at risk for invasive fungal disease. Pediatr. Blood Cancer *66*, e27734. https://doi.org/10.1002/pbc.27734.
3. Gwinn, M., MacCannell, D., and Armstrong, G.L. (2019). Next-Generation Sequencing of Infectious Pathogens. JAMA *321*, 893–894. https://doi.org/10.1001/jama.2018.21669.
4. ICH (2024). Q5A(R2) Viral Safety Evaluation of Biotechnology Products Derived from Cell Lines of Human or Animal Origin.
5. Bova, R.A., Diaz, L., Eubank, S., Overgard, A., Armstrong, A., and Hasson, B. (2024). Validation of a next generation sequencing method for adventitious virus detection: Demonstration of sensitivity in multiple cell lines. Biologicals *86*, 101771. https://doi.org/10.1016/j.biologicals.2024.101771.
6. Chin, P.J., Lambert, C., Beurdelay, P., Charlebois, R.L., Colinet, A.S., Eloit, M., Gilchrist, S., Gilleece, M., Hess, M., Leimbach, A., et al. (2025). Virus detection by short read high throughput sequencing in a high virus low cellular background. NPJ Vaccines *10*, 61. https://doi.org/10.1038/s41541-025-01104-1.
7. Beurdeley-Fehlbaum, P., Pennington, M., Hégerlé, N., Albert, M., Bennett, A., Cheval, J., Clark, A., Cruveiller, S., Desbrousses, C., Frederick, J., et al. (2023). Evaluation of a viral transcriptome Next Generation Sequencing assay as an alternative to animal assays for viral safety testing of cell substrates. Vaccine *41*, 5383–5391. https://doi.org/10.1016/j.vaccine.2023.07.019.
8. Brussel, A., Brack, K., Muth, E., Zirwes, R., Cheval, J., Hebert, C., Charpin, J.M., Marinaci, A., Flan, B., Ruppach, H., et al. (2019). Use of a new RNA next generation sequencing approach for the specific detection of virus infection in cells. Biologicals *59*, 29–36. https://doi.org/10.1016/j.biologicals.2019.03.008.
9. Ng, S., Braxton, C., Eloit, M., Feng, S., Fragnoud, R., Mallet, L., Mee, E., Sathiamoorthy, S., Vandeputte, O., and Khan, A. (2018). Current Perspectives on High-Throughput Sequencing (HTS) for Adventitious Virus Detection: Upstream Sample Processing and Library Preparation. Viruses *10*, 566. https://doi.org/10.3390/v10100566.
10. Teitz, S.B., Bailey, A., Blumel, J., Khan, A., Armstrong, A., Kreil, T.R., Hongo-Hirasaki, T., Leisi, R., Cetlin, D., Azimpour, C., et al. (2025). Viral Safety Reloaded-Insights from PDA's Virus Conference Around the Finalized ICH Q5A (R2) and NGS Workshop. PDA J. Pharm. Sci. Technol. *79*, 337–354. https://doi.org/10.5731/pdajpst.2025-000009.1.
11. Khan, A.S., Mallet, L., Blümel, J., Cassart, J.P., Knezevic, I., Ng, S.H.S., Wall, M., Jakava-Viljanen, M., Logvinoff, C., Goios, A., and Neels, P. (2023). Report of the third conference on next-generation sequencing for adventitious virus detection in biologics for humans and animals. Biologicals *83*, 101696. https://doi.org/10.1016/j.biologicals.2023.101696.
12. Kubacki, J., Fraefel, C., and Bachofen, C. (2021). Implementation of next-generation sequencing for virus identification in veterinary diagnostic laboratories. J. Vet. Diagn. Invest. *33*, 235–247. https://doi.org/10.1177/1040638720982630.
13. Hirai, T., Kataoka, K., Yuan, Y., Yusa, K., Sato, Y., Uchida, K., and Kono, K. (2024). Evaluation of next-generation sequencing performance for in vitro detection of viruses in biological products. Biologicals *85*, 101739. https://doi.org/10.1016/j.biologicals.2023.101739.
14. Rajagopala, S.V., Bakhoum, N.G., Pakala, S.B., Shilts, M.H., Rosas-Salazar, C., Mai, A., Boone, H.H., McHenry, R., Yooseph, S., Halasa, N., and Das, S.R. (2021). Metatranscriptomics to characterize respiratory virome, microbiome, and host response directly from clinical samples. Cell Rep. Methods *1*, 100091. https://doi.org/10.1016/j.crmeth.2021.100091.
15. EDQM (2026). Ph. Eur. Chapter 2.6.41. High-Throughput Sequencing for the Detection of Viral Extraneous Agents.
16. Ogunbayo, A.E., Sabiu, S., and Nyaga, M.M. (2023). Evaluation of extraction and enrichment methods for recovery of respiratory RNA viruses in a metagenomics approach. J. Virol. Methods *314*, 114677. https://doi.org/10.1016/j.jviromet.2023.114677.
17. Manso, C.F., Bibby, D.F., and Mbisa, J.L. (2017). Efficient and unbiased metagenomic recovery of RNA virus genomes from human plasma samples. Sci. Rep. *7*, 4173. https://doi.org/10.1038/s41598-017-02239-5.
18. Alberti, A., Belser, C., Engelen, S., Bertrand, L., Orvain, C., Brinas, L., Cruaud, C., Giraut, L., Da Silva, C., Firmo, C., et al. (2014). Comparison of library preparation methods reveals their impact on interpretation of metatranscriptomic data. BMC Genom. *15*, 912. https://doi.org/10.1186/1471-2164-15-912.
19. Kenney, S.P., and Meng, X.J. (2015). Identification and fine mapping of nuclear and nucleolar localization signals within the human ribosomal protein S17. PLoS One *10*, e0124396. https://doi.org/10.1371/journal.pone.0124396.
20. Shukla, P., Nguyen, H.T., Torian, U., Engle, R.E., Faulk, K., Dalton, H.R., Bendall, R.P., Keane, F.E., Purcell, R.H., and Emerson, S.U. (2011). Cross-species infections of cultured cells by hepatitis E virus and discovery of an infectious virus-host recombinant. Proc. Natl. Acad. Sci. USA *108*, 2438–2443. https://doi.org/10.1073/pnas.1018878108.
21. Liu, H., Liao, H.M., Li, B., Tsai, S., Hung, G.C., and Lo, S.C. (2019). Comparative Genomics, Infectivity and Cytopathogenicity of American Isolates of Zika Virus that Developed Persistent Infections in Human Embryonic Kidney (HEK293) Cells. Int. J. Mol. Sci. *20*, 3035. https://doi.org/10.3390/ijms20123035.
22. Li, B., Liao, H.M., Liu, H., Tsai, S., Zhang, J., Hung, G.C., Chin, P.J., Gao, Y., and Lo, S.C. (2018). Comparative genomics, infectivity and cytopathogenicity of Zika viruses produced by acutely and persistently infected human hematopoietic cell lines. PLoS One *13*, e0203331. https://doi.org/10.1371/journal.pone.0203331.
23. Fares-Gusmao, R., Jiang, Z., Subramaniam, S., Visser, B.J., Scott, A., Ishida, Y., Saito, T., Baylis, S.A., McGivern, D.R., Osiowy, C., et al. (2022). Development and characterization of secondary standards for nucleic acid amplification technology (NAAT) assays for detection of hepatitis E virus. J. Clin. Virol. *157*, 105325. https://doi.org/10.1016/j.jcv.2022.105325.
24. Li, H. (2013). Aligning sequence reads, clone sequences and assembly contigs with BWA-MEM. Preprint at arXiv. https://doi.org/10.48550/arXiv.1303.3997.
25. Kim, D., Paggi, J.M., Park, C., Bennett, C., and Salzberg, S.L. (2019). Graph-based genome alignment and genotyping with HISAT2 and HISAT-genotype. Nat. Biotechnol. *37*, 907–915. https://doi.org/10.1038/s41587-019-0201-4.
26. Robinson, J.T., Thorvaldsdóttir, H., Winckler, W., Guttman, M., Lander, E.S., Getz, G., and Mesirov, J.P. (2011). Integrative genomics viewer. Nat. Biotechnol. *29*, 24–26. https://doi.org/10.1038/nbt.1754.
27. Stang, A., Petrasch-Parwez, E., Brandt, S., Dermietzel, R., Meyer, H.E., Stühler, K., Liffers, S.T., Überla, K., and Grunwald, T. (2009). Unintended spread of a biosafety level 2 recombinant retrovirus. Retrovirology *6*, 86. https://doi.org/10.1186/1742-4690-6-86.
28. van Dijk, E.L., Auger, H., Jaszczyszyn, Y., and Thermes, C. (2014). Ten years of next-generation sequencing technology. Trends Genet. *30*, 418–426. https://doi.org/10.1016/j.tig.2014.07.001.

29. Khan AS, C.P., Fuentes, S.M., and Tsou, J.H.; Study Group (2024). A Collaborative Study to Evaluate the Proposed First WHO International Reference Panel for Adventitious Virus Detection in Biological Products by High-throughput Sequencing (HTS) Technologies (World Health Organization WHO/BS/2024.2471). https://cdn.who.int/media/docs/default-source/biologicals/call-for-comments/who_bs_2024.2471_1st-inter-ref-panel-for-adventitious-virus-detection_cber_khan–arifa.pdf?sfvrsn=e410a551_2.

30. Balm, M.N.D., Lee, C.K., Lee, H.K., Chiu, L., Koay, E.S.C., and Tang, J.W. (2012). A diagnostic polymerase chain reaction assay for Zika virus. J. Med. Virol. *84*, 1501–1505. https://doi.org/10.1002/jmv.23241.

31. Garson, J.A., Ferns, R.B., Grant, P.R., Ijaz, S., Nastouli, E., Szypulska, R., and Tedder, R.S. (2012). Minor groove binder modification of widely used TaqMan probe for hepatitis E virus reduces risk of false negative real-time PCR results. J. Virol. Methods *186*, 157–160. https://doi.org/10.1016/j.jviromet.2012.07.027.

32. Ewels, P., Magnusson, M., Lundin, S., and Käller, M. (2016). MultiQC: summarize analysis results for multiple tools and samples in a single report. Bioinformatics *32*, 3047–3048. https://doi.org/10.1093/bioinformatics/btw354.

33. Chen, S., Zhou, Y., Chen, Y., and Gu, J. (2018). fastp: an ultra-fast all-in-one FASTQ preprocessor. Bioinformatics *34*, i884–i890. https://doi.org/10.1093/bioinformatics/bty560.

34. Liao, Y., Smyth, G.K., and Shi, W. (2014). featureCounts: an efficient general purpose program for assigning sequence reads to genomic features. Bioinformatics *30*, 923–930. https://doi.org/10.1093/bioinformatics/btt656.

OMTA, Volume *34*

Supplemental information

Seeing the invisible: How RNA extraction and library prep shape viral read recovery in RNA-seq

Sayma Afroj, Binsheng Gong, Aaron Scholl, Bingjie Li, Joshua Xu, and Sandip De

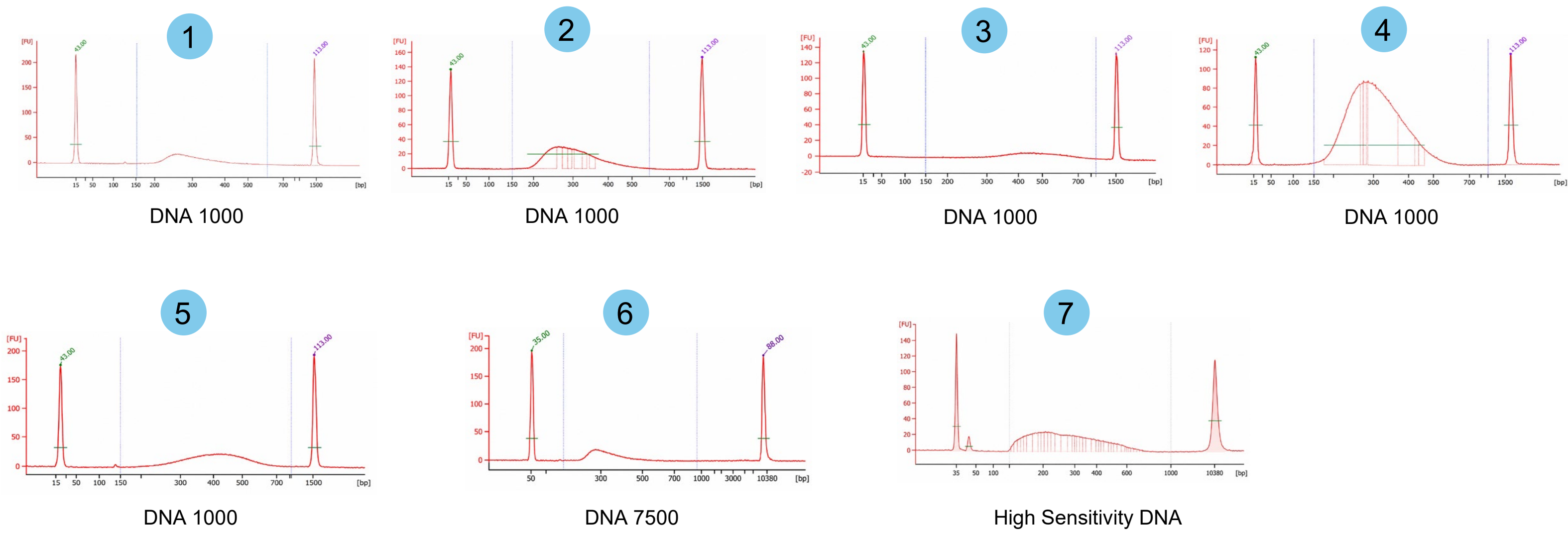

Fig S1. Representative electropherogram images for RNA-Seq libraries prepared using 7 different methods. Bioanalyzer DNA kits used for analyzing the library distribution is mentioned below.

		HEV-AVG	HEV-SE	RSV-AVG	RSV-SE	REO-AVG	REO-SE	ZIKV-AVG	ZIKV-SE	FeLV-AVG	FeLV-SE
	Control	0.000	0.000	0.062	0.022	0.013	0.013	*15.403*	*3.671*	0.000	0.000
1	Cocktail 1	0.191	0.037	2.946	0.027	0.271	0.071	*15.837*	*2.546*	0.060	0.005
	Cocktail 2	1.538	0.072	38.310	0.380	5.066	1.406	*37.308*	*2.555*	7.405	0.504
	Control	0.000	0.000	0.022	0.013	0.000	0.000	*0.710*	*0.061*	0.013	0.004
2	Cocktail 1	0.005	0.005	0.178	0.006	0.017	0.017	*0.781*	*0.132*	0.005	0.005
	Cocktail 2	0.132	0.009	3.075	0.010	0.251	0.022	*1.910*	*0.100*	0.993	0.035
	Control	0.000	0.000	0.000	0.000	0.000	0.000	0.000	0.000	0.000	0.000
3	Cocktail 1	0.186	0.070	7.170	0.110	0.138	0.003	0.525	0.199	0.152	0.075
	Cocktail 2	3.266	0.052	117.872	6.174	5.358	0.035	21.485	1.473	26.225	0.686
	Control	0.062	0.062	1.569	1.465	0.118	0.100	1.014	0.815	0.473	0.473
4	Cocktail 1	*0.000*	0.000	37.879	4.426	0.136	0.136	0.885	0.367	0.141	0.141
	Cocktail 2	*0.000*	0.000	658.374	11.662	1.078	0.057	5.479	2.407	2.419	0.086
	Control	0.004	0.004	0.128	0.128	0.000	0.000	0.000	0.000	0.000	0.000
5	Cocktail 1	0.013	0.000	54.850	17.272	0.007	0.007	0.562	0.550	*0.000*	0.000
	Cocktail 2	0.011	0.011	624.347	27.601	0.633	0.375	1.141	0.364	5.015	2.135
	Control	0.000	0.000	0.000	0.000	0.012	0.001	*31.068*	*2.549*	0.000	0.000
6	Cocktail 1	0.387	0.125	4.490	0.063	0.419	0.031	*27.348*	*4.926*	0.141	0.053
	Cocktail 2	2.723	0.219	49.944	3.463	4.099	0.394	*67.402*	*30.723*	16.322	1.700
	Control	0.000	0.000	0.000	0.000	0.000	0.000	0.000	0.000	0.000	0.000
7	Cocktail 1	0.048	0.048	1.178	0.431	0.024	0.024	0.127	0.042	0.023	0.001
	Cocktail 2	0.568	0.202	26.279	2.010	1.209	0.043	2.696	0.343	4.721	0.982

Fig S2. Mapped read counts (CPM) for HEV, Reovirus, RSV, ZIKV and FeLV across library prep conditions and spike-in groups (Control, Cocktail 1, Cocktail 2). Each library preparation method was performed in duplicate (n = 2). Cases where the standard error equals the mean (highlighted with blue boxes) arise when one replicate has a zero value and the other contains the full signal highlighting signals in control samples are stochastic single-replicate events.

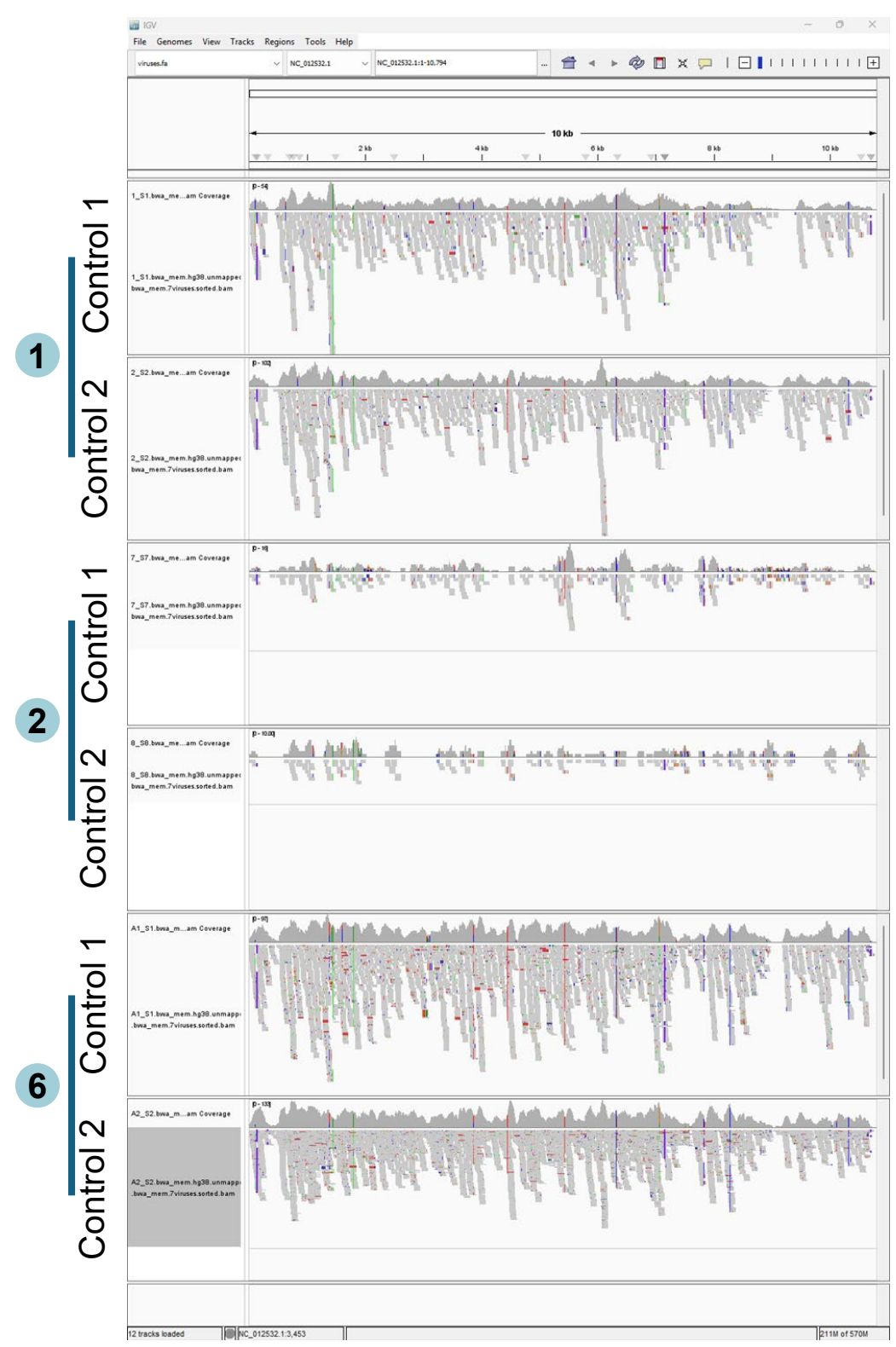

Fig S3. An Integrative Genomics Viewer (IGV) screenshot showing BAM file alignments from control samples generated using library preparation methods 1, 2, and 6.

Tables S1–S6

Table S1. RT-qPCR CT values for comparison of relative viral RNA recovery across RNA extraction methods and sample matrices. CT values for the indicated viruses following RNA extraction using the five evaluated methods. Ct values were used as comparative measures of relative viral RNA recovery within the same virus and sample matrix and were not used for absolute quantification or determination of extraction efficiency.

Table S2. Initial exploratory mapping analysis- RNA-seq read mapping results across library preparation methods and experimental conditions. We used a combined host + viruses genome for the analysis.

Table S3. Final host-filtered viral mapping- RNA-seq read mapping results across library preparation methods and experimental conditions. We used a combined host - viruses genome for the analysis.

Table S4. Competitive flavivirus re-alignment analysis of ZIKV-mapped reads detected in unspiked control samples. ZIKV-mapped reads from control samples were re-aligned against an expanded panel of closely related flaviviruses using paired-end and independent single-end alignment strategies to assess whether the unexpected ZIKV signal resulted from misclassification.

Table S5. Untargeted viral detection using the Reference Viral Database (RVDB), post host read filtering. Summary of viruses identified by the RVDB-based bioinformatics pipeline across library preparation methods and experimental conditions. Results represent screening-level bioinformatic hits and should not be interpreted as confirmed viral detections without additional confirmatory evaluation.

Table S6. RNA-seq library preparation and sequencing batch information. Summary of library preparation and sequencing batch information for samples sequenced using the Illumina Novaseq 6000 platform.
